# Genomic Mining of Phylogenetically Informative Nuclear Markers in Bark and Ambrosia Beetles

**DOI:** 10.1371/journal.pone.0163529

**Published:** 2016-09-26

**Authors:** Dario Pistone, Sigrid Mugu, Bjarte Henry Jordal

**Affiliations:** Department of Natural History, The University Museum, University of Bergen, PB7800, NO-5020 Bergen, Norway; Onderstepoort Veterinary Institute, SOUTH AFRICA

## Abstract

Deep level insect relationships are generally difficult to resolve, especially within taxa of the most diverse and species rich holometabolous orders. In beetles, the major diversity occurs in the Phytophaga, including charismatic groups such as leaf beetles, longhorn beetles and weevils. Bark and ambrosia beetles are wood boring weevils that contribute 12 percent of the diversity encountered in Curculionidae, one of the largest families of beetles with more than 50000 described species. Phylogenetic resolution in groups of Cretaceous age has proven particularly difficult and requires large quantity of data. In this study, we investigated 100 nuclear genes in order to select a number of markers with low evolutionary rates and high phylogenetic signal. A PCR screening using degenerate primers was applied to 26 different weevil species. We obtained sequences from 57 of the 100 targeted genes. Sequences from each nuclear marker were aligned and examined for detecting multiple copies, pseudogenes and introns. Phylogenetic informativeness (PI) and the capacity for reconstruction of previously established phylogenetic relationships were used as proxies for selecting a subset of the 57 amplified genes. Finally, we selected 16 markers suitable for large-scale phylogenetics of Scolytinae and related weevil taxa.

## Introduction

In the postgenomic era, obtaining well resolved and highly supported molecular phylogenies of hyper-diverse eukaryotic lineages continues to represent a major challenge. Previous attempts on investigating phylogenetic relationships in beetles have demonstrated recurrent problems in resolving deeper relationships such as those between the four beetle suborders, but also much younger divergences [1–4]. One of the most problematic groups includes the weevils, where the majority of tribes and subfamilies remain unresolved despite considerable efforts in assembling molecular data [5–8]. Bark and ambrosia beetles in the subfamily Scolytinae represent a weevil lineage where much effort has been invested in developing molecular markers for phylogenetic analysis [9, 10]. Nevertheless, resolution between many Cretaceous relationships remains rather low [11], emphasizing the scarceness of molecular markers to resolve this particular phylogeny.

So far, the vast majority of phylogenetic studies on beetles were based on markers such as ribosomal RNAs and mitochondrial cytochrome oxidase I and II genes [8, 12–15]. With the exception of nuclear ribosomal genes (*18s* and *28s rRNAs*) are most markers useful for the resolution of Cenozoic divergences, showing lack of phylogenetic signal for Cretaceous time frames [10]. In the last years, a growing number of phylogenetic studies on beetles have started to include nuclear protein coding genes, especially *EF-1α*, *CAD*, *ArgK*, and *wingless* [11, 16, 17], which are also widely used in other insect taxa [18–21]. However, a relatively limited amount of work has been done to discover and select additional nuclear genes for beetle systematics [22, 23–25], and all studies to date were based on less than 10 molecular markers [26, 27]. Therefore, obtaining a high degree of phylogenetic resolution in beetles is difficult; a direct consequence of high species diversity and a limited number of informative markers.

The first studies on the utility of protein coding genes in insect systematics date back to more than 20 years ago [28–30]. The advancement of insect phylogenies has largely been driven by the development of new markers in Lepidoptera [31]. At present, dozens of nuclear markers can be chosen to investigate Lepidoptera phylogeny at various ranks [18, 32–36]. Hymenoptera is another group where a consistent number of nuclear markers have been developed [37–39]. Although similar studies have been carried out in other insect groups such as Diptera [40–42], the majority of the remaining insect orders present a situation more similar to Coleoptera with few published markers conserved across different families [43, 44]. Thus, increasing the number of phylogenetic characters from protein coding nuclear genes is of mandatory importance for achieving robust phylogenetic hypotheses in beetle systematics.

Recently, the advent of next generation sequencing (NGS) technologies has contributed to additional ground-breaking advancements in the systematics field, profoundly increasing the level of resolution compared to previous phylogenies based on single or few genes [45]. Genomic and transcriptomic data obtained from NGS based research has led to predictive insect phylogenies, which now more clearly reveal key events in insect evolutionary history [46–50]. New developments based on ultra-conserved elements (UCEs) or RAD-sequencing will increase resolution also at lower taxonomic ranks in insects [51, 52]. However, the benefits of NGS are generally counterbalanced by the high cost and computationally demanding analyses of such high throughput data. The utility of few well-characterized markers should not be underestimated as they represent a rapid and cost effective approach for resolving small scale phylogenies.

Bark and ambrosia beetles in the subfamily Scolytinae constitute a group of highly derived, small wood boring weevils capable of excavating galleries into different parts of dead trees, shrubs and bushes, as well as in lianas and other plant tissues in different forest habitats throughout the world [53]. Scolytinae is generally regarded as a well-supported clade of more than 6000 described species representing approximately 12 percent of the entire diversity in the family Curculionidae [5, 54, 55]. A tremendous variability in life cycles, reproductive strategies, mating systems, host plants interactions, feeding behavior and ecology has been documented [56, 57], which makes this group of beetles particularly interesting to study in a phylogenetically comparative context. Phylogenies of Scolytinae have so far relied on a combination of five molecular markers (one mitochondrial and four nuclear genes) and eventually morphological characters. Given the high diversity of Scolytine species, additional data are needed to obtain sufficient resolution at deeper nodes.

In order to select new phylogenetic markers, 100 different nuclear genes were screened by PCR using degenerate primers and tested in a restricted but representative group of Scolytinae and other weevils. With the aim of developing slowly evolving genes, the properties of each gene fragment were evaluated based on PCR amplification and sequencing success and their phylogenetic performance. This study reports on the development and utility of 16 novel markers for weevils, with a particular focus on bark and ambrosia beetles in the subfamily Scolytinae.

## Materials and Methods

We included 18 species of bark and ambrosia beetles and 8 additional weevils from other subfamilies for primer screening (Table 1 and S1 Table). These beetles were collected by one of the authors (BHJ) during fieldwork in tropical forests (1998–2012). Collection permits were requested from authorities in Uganda, Tanzania, Cameroun, South Africa and Madagascar. Ethical guidelines were followed. Voucher specimens are deposited in the Coleoptera collection of the University Museum of Bergen, University of Bergen, Norway. All weevils, Platypodinae and Scolytinae species used in this study were previously described in other phylogenetic studies [7, 11, 58].

**Table 1:** Weevil species included in this study.

Species	Code	Subfamily	Tribe	Country
Brentidae sp.	BrBre05	Brentidae (familiy)	Brentinae	Cameroon
*Mesites fusiformis*	CsMes01	Cossoninae	Cossonini	Spain
*Pselactus sp*.	CsPse01	Cossoninae	Onycholipini	Portugal (Madeira)
*Larinus sp*.	ClLar01	Lixinae	Cleonini	Russia
*Porthetes hispidus*	MoPor01	Molytinae	Amorphocerini	South-Africa
*Platypus impressus*	PlPla07	Platypodinae	Platypodini	Tanzania
*Triozastus marshalli*	PlTri02	Platypodinae	Platypodini	Cameroon
*Chaetastus tuberculatus*	TsCha02	Platypodinae	Tesserocerini	Cameroon
*Pityophthorus micrographus*	CoPit01	Scolytinae	Corthylini	Norway
*Diamerus inermis / D*. *hispidus*	DiDia03 / DiDia04	Scolytinae	Diamerini	Tanzania / Madagascar
*Dryocoetes autographus*	DrDry01	Scolytinae	Dryocoetini	Russia
*Ozopemon uniseriatus*	DrOzo02	Scolytinae	Dryocoetini	Papua New Guinea
*Hylastes attenuatus*	HtHyt06	Scolytinae	Hylastini	Sweden
*Hylesinus varius*	HlHyl02	Scolytinae	Hylesinini	Sweden
*Kissophagus hederae*	HlKis01	Scolytinae	Hylesinini	Austria
*Chaetoptelius vestitus*	ToCha01	Scolytinae	Hylurgini	Morocco
*Dendroctonus terebrans / D*. *micans*	ToDen02 / ToDen01	Scolytinae	Hylurgini	USA
*Tomicus piniperda*	ToTom01	Scolytinae	Hylurgini	Norway
*Acanthotomicus sp*.	IpAca01	Scolytinae	Ipini	Cameroon
*Pityogenes quadridens*	IpPit03	Scolytinae	Ipini	Sweden
*Premnobius cavipennis*	PrPre01	Scolytinae	Premnobiini	Sierra Leone
*Camptocerus aenipennis*	ScCam02	Scolytinae	Scolytini	Guyana
*Cnemonyx vismiacolens*	ScCne01	Scolytinae	Scolytini	Guyana
*Scolytus intricatus*	ScScl02	Scolytinae	Scolytini	Czech Republic
*Xyleborus affinis*	XyXyl00	Scolytinae	Xyleborini	Cameroon
*Xyleborus monographus*	XyXyl03	Scolytinae	Xyleborini	Czech Republic

Degenerate primers were designed on conserved regions in the alignment of insect nucleotide sequences that were available from genomic and transcriptomic sources. Two or more consecutive degenerate sites were preferentially avoided as well as the use of completely degenerate sites (N). A total of 274 primers were designed (Table 2 - only successful primers reported).

The procedure for primer selection can be summarized as follows: 1) putatively single copy expressed sequence tags (ESTs) longer than 800 base pairs were selected in GenBank for two different beetle species, *Tribolium castaneum* and *Dendroctonus ponderosae*; 2) preliminary BLAST searches were performed to discard unsuitable markers, based on the evidence for multiple paralogous copies (e.g. large gene families) or ambiguous genomic characterization (e.g. similar matching values for different proteins); 3) available sequences for each selected gene were aligned, including annotated genomic and transcriptomic sequences from model organisms (e.g. *Drosophila melanogaster*, *Apis mellifera* and *Bombyx mori*) to determine intron-exon structure; 4) degenerate primers were designed; 5) a PCR screening was run and products with the expected correct size (albeit highly variable due to presence of introns) were sequenced; 6) markers reaching a minimum PCR and sequencing success of 20% were used to reconstruct single gene phylogenies (Bayesian) and trees were compared to previously established and well-supported clades [5, 7, 10, 11].

DNA was extracted from individual specimens using DNeasy Blood & Tissue kit (Qiagen) following the manufacturer’s instructions. The PCR reaction mixture contained 2.5 μl 10x PCR buffer (Qiagen), in which the final concentration of MgCl_2_ was 2.0 mM, 200 μM of each dNTP (Sigma Aldrich), 0.5 μM of each primer, 0.125 units Hot Start Taq^®^ DNA polymerase (Qiagen), 2 μl DNA, with water added to a final volume of 25 μl. A negative control (sterile water) was included in each test. The PCR was performed using a S1000^™^ Thermal Cycler (BIO-RAD Laboratories, Inc.). Three standard cycle programs were used for the initial screening: denaturation step at 95°C for 5 minutes, 35 cycles of 30 seconds at 95°C, 30 seconds at 48, 52 and 58°C, 60 seconds at 72°C, and finally 5 minutes extension at 72°C. Further optimization included a gradient of annealing temperatures in the range of 44–62°C, modulating the extension time depending on the expected PCR product length, and MgCl_2_ concentration. We also considered two different touch-down PCR protocols for two of these genes (see Table 2 for details).

**Table 2:** Primer sequences and annealing temperature for the nuclear markers selected in this study. Furthermore, primers for additional genes for lower level phylogenetics are reported.

Gene acronym	Primer forward (5'-3')	Primer reverse (5'-3')	Annealing T°C
*EF2*	CGTTTCTAYGCBTTYGGHCGTG	CCYTCYTTRGTGGCCCAYTGG	TD 58 (10 cy) 44 (25cy)
	ATGATGGGYCGTTAYGTWGARGC		TD 58 (10 cy) 44 (25cy)
*Hsp70*	CAAGCYGACATGAAGCAYTGGCC	CGGGTGATGGAGGTGTAGAARTC	58
	GAYGGTATCTTYGARGTMAAGTC	CGRCCYTTGTCRTTRGTGATGG	55
*CCNC*	ATGGCTGGMAAYTTTTGGCARAG	TCGAGCAGATARAAYTCRCAYTC	52
*HDAC Rpd3*	ATGAARCCSCACMGSATAMGSATGAC	GTAGTCGTTRTARGGSAGYTCRTTGGC	53
		GCCACSGAAGTYTCRTASGTCCA	53/50
*Arr2*	CGYGARGAGGAYGARGTYATGGG	ACCATSGTRACYTCGCAATGYTGCAC	52
		CTCAAARACKATRTTGTCGTCRTCGTC	52
*Iap2*	TGGAAYTAYGGRGACCAAGTRATGGC	CCATCKGGCRTGYTCYGTCCAWGGATC	52
*PABP1*	CCRATTCGYATYATGTGGTC	GAARGCRACAAAWCCRAAWCC	50
*Prp1*	ATGTCSGCKACTYTRGAYGCWGG	GGRTASGTGTTRTCYTGCATYTC	44
*CTR9*	GAAGGYGATAARATGGAWCARGC	TCGAAACAYTGKGCKGCATTTTC	52
*RCC1*	GGKTGYAATGACGARGGSGC	CGGCCCAATTGTCCYTGYTC	52
*SOD1*	TCCACATYCAYGARTTYGGGG	CCTTKKCCCAAATCATCMGG	TD 52 (10 cy) 46 (25cy)
*TPI*	CGHAAATTCGTWGTYGGWGGHAACTGG	CKGARCCYCCRTATTGRATTC	50
	GGTGGHAACTGGAARATGAACGG		52
*ADA2*	GAYATGYTDGAYGTVCATGC	ACAGGRCCRGCTTCRCCRCAATG	52
	AARTTYAATGCCAAATAYAAYCC	GGWCCRGCTTCACCRCARTGWGG	48/52
*UBA5*	TTGGKAGYGTAACWGCRGAAATG	ATATGGCCWGARACSGCRTTTTC	52
*Cda4*	TACGARGARTGGGTKGGRGARATG	AACCAATTMGTRTGRAASGGCATC	48
*FEN1*	GARGCCCCYTGYGARGCKGARGC	TCACCATGCCYTCYTCRTCMGG	48
*ACTB*	CTGAAGCCCCMTTGAACCCMAAGGC	GAGATCCACATCTGYTGGAARGTGG	
*CXorf56*	GAAGYATTGCRTGTTCSGAYAC	GTCACMGAACTGAAYTTKCCC	
*eRF1*	GTTGGCAGATGAATTTGGAACRGC	CCRAABAGAGCTCCRTTACCATCC	
*U2AF*	ATYGCTGGATTWAAYGGRATGC	TCTCKTCTRTGRTACTTRTCSGGWTC	
*MAD*	YAAYTTYCCWGCYATGRTWCC	ACACCRTGRTTYTTWGCWCC	
*mp20*	GACAAGGARGCCCARGARTGGATCG	TCCCACAGRTCAACTGTYTGGAARAC	
		GGTCCGGGCCCAYTCRGRGTGCYTGTTAGG	
*5MP*	CATGACKTTTATGMGKGCKTTC	CTTCYTCRGCGTTTTGWAGCC	
*Pi4k*	TGYTGYCCKTGYTGYTTYGG	TGGTAYGGRTASGCYCGCC	
*Gel*	GAYGAGGGCSGGWTCSGCWGC	AGGATRAAGCARTCRCCTTTGTTC	
*C1-THF*	CATYTRACYGGYGAYATYCATGC	ACAGCYCCYGTKGCYCCCAAATC	
*alpha-Spec*	CAYGCHAATGCWTTCCATCARTGG	GGYTGKCCYTCYTCWACCATYGG	
*AATS*	CATCAYACGTTTTTTGAGATG	GCATGRTCNGCTAARACNCGRTARGCC	
*Hsp90*	GATCATCAATATSTTCTACTC	TCTCCGGTGATGWARTAGATG	
*dldE3*	GGRGAYTGTATWCATGGRCC	GCYTCRTTRATBARTTCRCC	
	CATCCWGAAGTKGGMTGGGTKGG		
*Mpgt*	AAACCSCTGTTYCCMGTTGCKGG	GCMGTTTTYAACTGSGACCACC	
*NaK*	GGYGGTTTCGCSWTGYTGYGTGGATCGG	GCGACGATGATACCGATCARGAAGATGACAGC	
*Fbox11*	AATGCWTTRGCTGGWATYTGGG	CCRCCRTGYTGACCRTGRTG	
*UDE*	AAGCCRGACACCGTWCCCGG	CTGGCWTCRGGRCTGTACGCCC	
*GTPbp*	ATTARAAYGTAKCCATCGTTRCCCC	GTGTTGATAATWGASGACTTGCC	
*CatL*	CACATTTACACTTTYAACCCRATG	ACCARCTGTTYTTMACCARCCAGTA	
*TpC*	CTTCCCSCMGARCARATYGCCG	CCTCSCCRGTCATCATCTCCATG	
*PGI*	GGCCCSCTKATGGTRACCGAAGC	CCCAGCTCCACKCCCCATTGGTC	
*AcCoA*	GGTGTACTGCKGAYATTGGYTGGATCAC	GGAAACSCAGCMGCKCCWGGYTTCAT	
		CATCAGRTGYCCKGASACGTTYARCAT	
*Ucdk*	GAGCACKGTWTGCAARCGYATWATGG	CCYCTWGGAATRATRACATCAGC	
*PPO1*	AAYCTSCACCAYTGGCAYTGGC	CGGAASGTSCKCTCRAASGG	
*Prp6*	AATCCSAATCATCCWCCGGCKTGG	TTCTTCCAGYTTRGCSGCRGTWGTCC	
*Mxp*	TAMGSACRGCSTAYACSAACAC	CGCTTGTGYTTCATSCKCCG	
*Npl4*	CTCGYTGYGTSCAYTGCTC	TCGCGCACYAGCGCCATRCAYTG	
*Cam1*	GAYGGMGATGGCACRATYACTACC	TCRTAATTGACCTGACCGTCRCC	
*STX1A*	ATGACYAARGAYAGATTRGCRGC	GCCATRTCCATRAACATRTCRTG	
*TP120b*	TWGGRAATGTCAAYGTYTC	AAGCTCAACCCKCKCCACATCC	
*CHS1*	CATATMTTYTTCGAYGAYGC	CAACGATCYTCKCCYTGATC	
*DDX49*	AARGCTATACGARGAYCCWTATGG	TGCCTGCYCTAGCWGTYCTYCC	
*GTF2H3*	CTCGCATTTGATGCAGAAGGC	CARATYGGRCTAAACTTGCA	
*IF3*	ACTCGCTYTACAAAATGTTGGG	CTTTSGTRTCGGCRATATGRATC	
*TIF6*	GACACRATWCCSGTGGTSCATGC	CTACCWCARTTWACYGTTCC	
*IDH*	TACAAYGTWGGAATWAARTGTGC	CAMACAAARCCYCCYTCMGATTTC	
*Ecr*	GAAGTKATGATGTTCMGRATGGC	GAWGCACATYTCDGARTTYTG	

PCR products were sequenced with the same primers as those used for amplification. DNA sequences of both strands were obtained using the BigDye Terminator cycle sequencing ready reaction kit (Applied Biosystems Inc.) using an automated DNA sequencer (Applied Biosystems Prism 3700) following the manufacturer’s instructions.

All obtained sequences were submitted to BLAST analyses, accepting a correct gene target if the cutoff value was below 1E-4. All sequences for each gene were aligned with other insect sequences for a preliminary NJ analysis in PAUP* 4.0 [59] to detect deviant sequences. The sequences were checked by eye and using Bioedit 7.2.5 [60] and MAFFT [61] to align gene fragments with complex structure, caused either by to the presence of indels of coding triplets, or less frequently by long introns marked by unusual exon-intron borders such as the most common alternative splice site GC—AG [62].

Introns were trimmed and the coding fragments were translated into amino acid sequences using Bioedit 7.2.5 to check for translational errors (stop codons). All these preliminary analyses had the purpose of detecting pseudogenes or early signs of possible paralogs (e.g. high degree of amino acid substitutions). In addition, the amino acid sequences of the selected markers were examined in OrthoDB v9 to assess gene orthology [63, 64]. The orthology for each gene was confirmed by cluster of orthologous groups (COGs) comparison among arthropod sequences in the database. Ambiguous nucleotide positions in the coding region that were difficult to align were tentatively excluded (in *Arr2* and *Iap2*) to create an alternative alignment for comparisons (see results and discussion).

Phylogenetic analyses were performed on unambiguously aligned sequences obtained from a minimum of 5 species. Phylogenetic inference was based on Bayesian and maximum parsimony analyses, the latter as implemented in PAUP* 4.0. Node support in the parsimony analyses was estimated by bootstrap analyses using 20 random additions of heuristic searches for each of 200 bootstrap replicates. Bayesian phylogenetic analyses were performed in MrBayes 3.2 [65]. The most appropriate model for base frequencies and substitution rates was determined by jModelTest [66], using the Akaike information criterion (AIC). MrBayes searches were run for each gene separately and for concatenated datasets (8109 bp– 2702 aa) using the suggested models for each gene partition and a mixed model for amino acid substitution. In both cases, the search consisted of 2000000 generations with two independent runs, each with four simultaneous chains, and trees sampled every 1000 generations. The convergence diagnostics (SDSF, PSRF) and parameter sample plots were evaluated using the software Tracer 1.6 [67].

**Fig 1:**
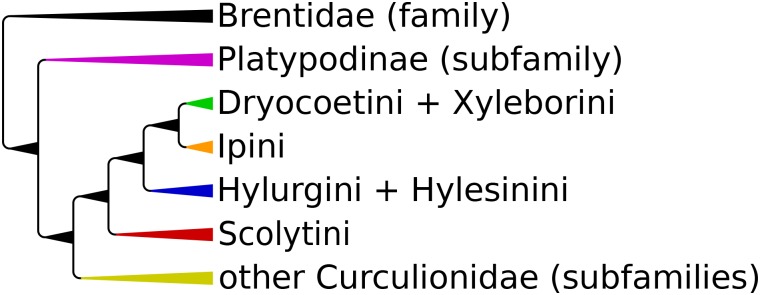
Schematic tree showing well supported relationships between tribes within the subfamily Scolytinae and other weevil families and subfamilies considered in this study.

An indirect measure of the phylogenetic signal in each marker was assessed through topological congruence with previously well documented clades [5–7, 10, 11, 68] which were used to derive a scheme of the current classification of Curculionoidea (Fig 1). These clades belong to six tribes of Scolytinae (A = Dryocoetini including Xyleborini, B = Ipini, C = Hylurgini + Hylesinini, D = Scolytini) and the subfamily Platypodinae (E). Rooting of the trees was dependent on the sequences available, and used in the following order: 1) Brentidae, 2) Platypodinae, 3) Cossoninae, Molytinae and Lixinae, 4) Scolytini [5, 6].

Basic properties of each gene, including the overall mean divergence of sequences (*p*-distance) and the variation in first, second and third positions, were calculated for each gene fragment using MEGA 6.0 [69]. Parsimony informative sites were calculated together with the homoplasy and retention indices (respectively HI and RI–S2 Table) using PAUP* 4.0. A phylogenetic informativeness profile (PI) was obtained for each gene using PhyDesign [70], an on-line program developed from a previous study [71]. Substitution rates for each position were calculated using HyPhy implemented in PhyDesign, selecting a K2P model (base frequencies = 0.25, transitions = 2, transversions = 1). The input time tree was obtained using Beast v1.8.2 [72], with topology constraints following previously published phylogenies of weevils and Scolytinae [5, 6, 11]. The tree was reconstructed using a concatenated dataset of 16 genes, using a GTR+I+ Γ model for each gene partition, and a Yule speciation process. We selected an uncorrelated lognormal relaxed molecular clock and used default priors as suggested by the authors (see XML S1 file in Supplementary information). Two calibration points were used: 116 Ma for the node subtending Scolytinae and other weevil subfamilies, and 30 Ma for clade A (Dryocoetini+Xyleborini).

## Results

Sequences were obtained for 57 different genes, whereas 43 primer sets never amplified the correct gene. A total of 798 sequences were obtained, but only 510 of these (64%) were unambiguously characterized as beetle orthologs in BLASTN search. Among the remaining 288 sequences, 53 were identified as non-beetle sequences (mainly from bacteria, fungi or nematodes associated with beetles) with different degree of confidence in gene identity. The remaining 235 sequences resulted in unreadable or poor quality sequences without a clear match in GenBank (E value > 1E-4, query coverage < 30% and/or less than 30% identity).

The evaluation of the 57 markers with readable sequences was based on the number of sequences obtained and their phylogenetic performance. When only one or two sequences were obtained for a gene (e.g. *cathepsin L*, *troponin C*, *acetyl coenzima A synthetase*, *maxillopedia*, *calmodulin 1*), the phylogenetic utility was not possible to assess. Other excluded markers produced a higher number of sequences, such as *odorant binding protein* (8 sequences) and *glycoside hydrolase family 31* (11), but these were largely unalignable. Another group of failed markers produced sequences from non-target organisms, such as *6-phosphogluconate dehydrogenase* of fungi, or *phosphoglucose isomerase* of bacteria. A total of 23 genes were discarded due to low amplification rates, high levels of non-beetle amplification, or generally low degree of gene orthology.

The remaining 34 genes showed differing degree of PCR and sequencing success (from 5 to 26 sequences obtained), and were further evaluated based on their capacity to recover known relationships at various taxonomic levels. Eighteen of these markers were found insufficiently informative for higher level phylogenetics, because no more than two of the predefined clades were reconstructed correctly. However, most discarded markers nevertheless revealed some phylogenetic utility at lower taxonomic level; including populations (see S3 Table for further details).

**Fig 2:**
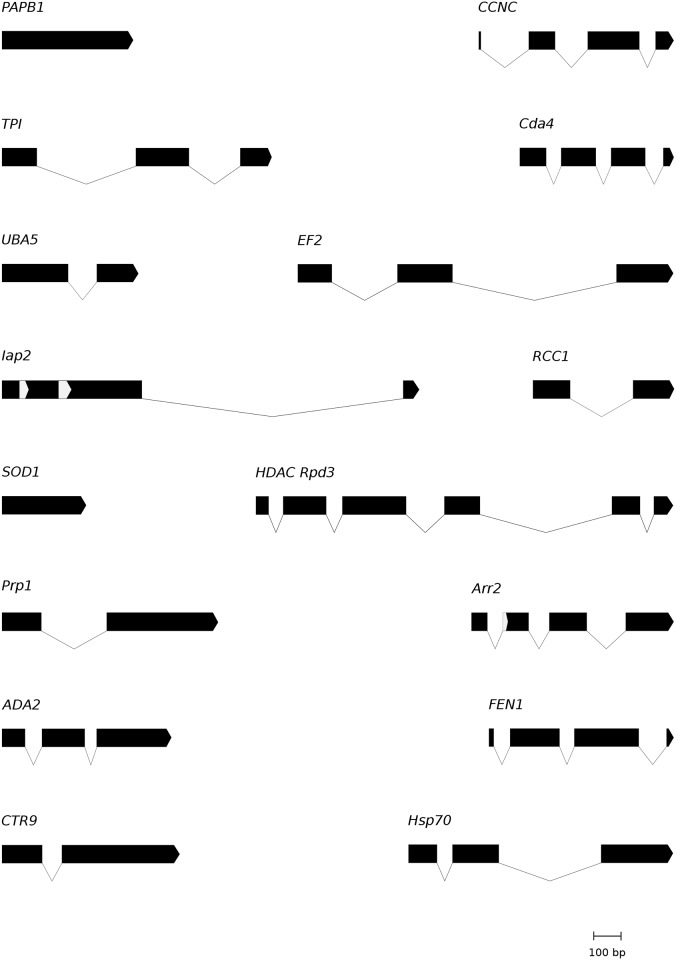
Structure of the PCR amplified gene fragments. The graphics illustrate intron-exon patterns in 16 markers with coding regions shown as black bars and introns as thin black lines. Length variable coding regions (indels) were colored in light grey (*Iap2* and *Arr2*).

We selected 16 genes that revealed a relatively high and stable PCR and sequencing success (from 50 to 100%) as the best candidates for Scolytinae phylogenetics (Table 3). All the verified sequences obtained in this study were deposited in GenBank database under the accession numbers KX160539—KX160803 (S1 Table). The species *Xyleborus affinis* was the most successful in PCR and sequencing (15 out of 16 possible sequences obtained); the other samples varied considerably in this respect with only 4 sequences obtained for *Larinus* sp. (S1 Table). The total fragment length, the presence of length-variable regions, and the number and position of introns, were mapped on the annotated genomes of *T*. *castaneum* and *D*. *ponderosae* (eventually transcriptomic and genomic data of other insect species) to create a map of the gene structure (Fig 2; see also Table 4).

**Table 3:** PCR and sequencing success for 16 selected genes.

GENE ACRONYM	A	B	C	D	E	F	G	H	Total (%)
***PABP1***	4	3	6	3	3	4	2	1	26 (100%)
***TPI***	4	2	6	-	2	2	2	-	18 (69%)
***UBA5***	3	3	5	3	2	2	1	1	20 (77%)
***Iap2***	3	3	1	2	1	4	2	-	16 (62%)
***SOD1***	2	1	4	3	2	3	1	-	16 (62%)
***Prp1***	3	3	5	1	3	1	2	-	18 (69%)
***ADA2***	3	2	2	2	3	-	2	-	14 (54%)
***CTR9***	2	2	4	2	-	1	2	-	13 (50%)
***CCNC***	4	2	5	2	2	2	2	1	20 (77%)
***Cda4***	2	1	4	-	3	1	1	1	13 (50%)
***HDAC Rpd3***	3	1	4	-	2	2	1	-	13 (50%)
***Arr2***	4	2	4	3	3	2	2	-	20 (77%)
***FEN1***	3	2	4	2	1	-	2	1	15 (58%)
***EF2***	2	2	3	2	3	-	2	-	14 (54%)
***Hsp70***	1	1	5	2	1	1	2	1	14 (54%)
***RCC1***	2	2	4	-	2	2	1	-	13 (50%)

The number of sequences obtained was reported for the following groups: A = Xyleborini + Dryocoetini, B = Ipini, C = Hylurgini + Hylesinini, D = Scolytini, E = Platypodinae, F = other Curculionidae subfamilies, G = other Scolytinae, H = Brentidae.

**Table 4:** Gene information.

Acronym	nucs	aa	Intron	Intron range (per intron)
***PABP1***	435	145	0	-
***TPI***	547	182	0–2	(457–51)(237–48)
***UBA5***	348	116	1	(94–48)
***Iap2***	672*	224*	1	(1131–50)
***SOD1***	213	71	0	-
***Prp1***	582	194	0–1	(258–55)
***ADA2***	624	208	2	(70–39) (105–53)
***CTR9***	627	209	0–1	(81–59)
***CCNC***	384	128	3	(200–69)(134–49)(71–58)
***Cda4***	410	136	0–3	(68–51)(63–56)(53)
***HDAC Rpd3***	858	286	3–5	(69–53)(70–54)(165–48)(564–54)(66–55)
***Arr2***	501*	167*	0–3	(110–51)(84–53)(158–55)
***FEN1***	417	139	1–3	(63–46)(55–42)(93–44)
***EF2***	621	207	1–2	(398–183)(702–84)
***Hsp70***	567	189	0–2	(61-?)(317–187)
***RCC1***	303	101	0–1	(250–51)

For each marker, the length of the sequenced coding region is given as the number of nucleotides and amino acids, together with the number and length of intron(s). The symbol * indicates genes with sequence length variability due to exonic indels.

OrthoDB analyses showed that 12 out of 16 genes selected in this study are present in single copy in more than 70% of the arthropod species currently in the database (133). *PABP1* and *UBA5* are in single copy in 96% of these species, followed by *HDAC Rpd3* (95%), *CCNC* (94%), *Prp1* (92%), *TPI*, *CTR9* and *FEN1* (90%), *Cda4* (89%), *EF2* (84%), *RCC1* (81%) and *ADA2* (74%). Only five genes are frequently in multi-copy status in arthropod genomes: *Hsp70* (single copy only in 2% of the species in the database), *Arr2* (4.5%), *Iap2* (8.3%) and *SOD1* (22%).

The best evolutionary model for the majority of the genes was GTR+I+Γ, except for *SOD1* and *Iap2* in which SYM+I+Γ and GTR+Γ were selected. Bayesian analysis of the concatenated nucleotide and amino acid data from 16 genes showed a well resolved tree topology (S1 Fig) with all expected clades recovered with maximum support, except Scolytini (pp = 0.75). The overall tree topology was correct with the exception of four weevil species that were nested inside Scolytinae as the sister lineage to Hylurgini (weakly supported in the amino acid analysis). Parsimony analyses of the concatenated dataset revealed similar results both for the nucleotide and amino acid datasets, with all major clades recovered with medium to high bootstrap support. However, the sub-family Scolytinae was not monophyletic in respect to the other advanced weevil species (S2 Fig).

**Fig 3:**
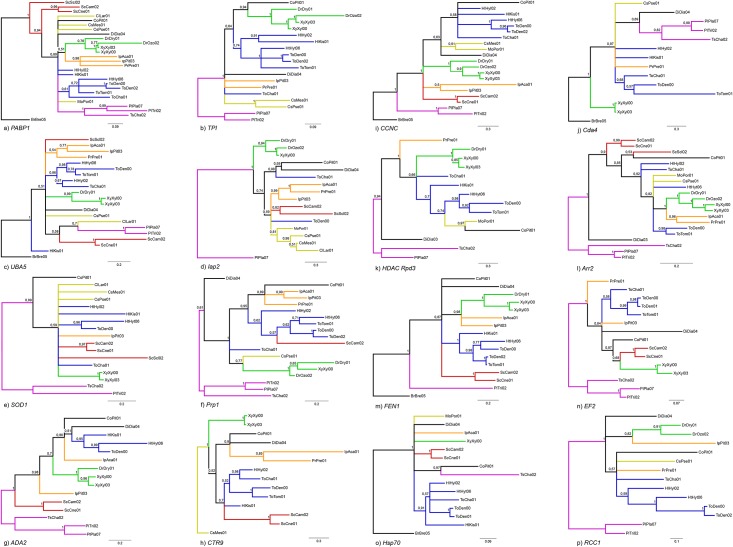
Phylogenetic trees based on Bayesian analyses of 16 selected genes. Trees were rooted with the most distant outgroup available for each marker. Posterior probabilities are given to the left of the nodes. Sequences of *D*. *ponderosae* (ToDen00) were obtained from GenBank.

Single gene analyses resulted in partially resolved phylogenies, mainly recovering a monophyletic Scolytinae, the majority of the predefined subgroups of Scolytinae (A-B-C-D), and the subfamily Platypodinae (Fig 3). All selected genes enabled the correct reconstruction of the most recent clade (A), with 3 genes obtaining the correct sister group (B). None of the selected genes showed high degree of incongruence that received high node support. Overall mean divergence in nucleotide sequences was reported for each codon position for each gene (S3 Fig).

### Selected genes for Scolytinae phylogeny

#### Polyadenylate binding protein 1 (PABP1)

*PABP1* was the most successful marker, with sequences obtained from all 26 species. The amplified fragment was 435 bp long, contained no introns, and translated into 145 amino acids. The phylogenetic analyses recovered almost all pre-defined clades (Fig 3a), but only two of them were highly supported (B, pp = 0.98; E, pp = 1). The tribe Scolytini was placed outside a polytomy including the remaining species of Scolytinae, the subfamily Platypodinae and the various other weevil subfamilies. No clear evidence of paralogs emerged from the analyses. Preliminary studies indicated increased phylogenetic performance with broader taxon coverage.

#### Triose-phosphate isomerase (TPI)

A combination of two primer pairs (two forward, one reverse) resulted in 67% PCR amplification and sequencing success. The aligned fragments consisted of 547 bp after removal of introns, which translated into 182 amino acids. Two introns were located in this gene fragment (Fig 2, Table 4). The phylogeny based on this marker confirmed the monophyly of Platypodinae (pp = 1), while Scolytinae formed a large polytomy including two advanced weevil species. Furthermore was Cossoninae monophyletic (pp = 1), in addition to one scolytine subgroup (A, pp = 1), and subgroup C almost so (Fig 3b).

#### Ubiquitin-like modifier activating enzyme 5 (UBA5)

The *UBA5* gene fragment is 348 bp long and translated into 116 amino acids. It was amplified from 20 different species (77%) in all main clades and contained one short intron in all species. The phylogeny recovered the monophyly of clades A and E with high node support (pp = 0.99 and 1, respectively) while clade D (pp = 1) had *Scolytus intricatus* excluded. Clade B and C were weakly supported (pp<0.95) and *Kissophagus hederae* was not included in Hylurgini (Fig 3c).

#### Inhibitor of apoptosis 2 (Iap2)

A total of 16 sequences (62%) were obtained from partial *Iap2*. This gene was amplified for only one species in Hylurgini (*Chaetoptelius vestitus*). The amplified fragments contained one long intron and a coding region of variable length up to 672 bp. Two hypervariable regions in the first exon were characterized by a series of indels of up to a maximum of six and ten triplets, respectively, consisting of serine-rich strings of amino acids. The intron range is within 50–80 bp in the majority of the species, but *D*. *ponderosae* (obtained from GenBank) contained a very long intron (1131 bp). BLASTN search indicated that a baculoviral Iap repeat is located between the two hypervariable regions. The phylogenetic analyses resulted in four monophyletic groups (clade A, pp = 0.94; clade B, pp = 0.99; D and F, pp<0.95), with no phylogenetic evidence of paralogs (Fig 3d).

#### Cu-Zn superoxide dismutase 1 (SOD1)

We amplified a short fragment (213 bp) of the cytoplasmic copper/zinc superoxide dismutase (*SOD1*), which contained no intron. We obtained 14 orthologous beetles sequences (54%) and five non-beetle sequences, but also amplified other genomic regions, suggesting non-specificity for this primer pair. The phylogeny contained several polytomies, with only one clade (A) receiving maximum support. Two internal nodes in the C and D clades were also recovered (pp>0.95). The tree was rooted with a monophyletic Platypodinae (Fig 3e).

#### Pre-mRNA-splicing factor ATP-dependent RNA helicase PRP1 (Prp1)

A fragment of the *Prp1* gene with the length of 582 bp (intron excised) corresponding to 194 amino acids, was amplified from 18 different species (70%). The presence of a single intron was observed in the majority of the species except three unrelated Scolytinae species and one Platypodinae. The phylogeny revealed two monophyletic groups (A, pp = 1; E, pp<0.95) and three groups which contained highly supported internal nodes (B, C and E), and a series of weakly supported incongruent relationships (Fig 3f). The tree was rooted on a monophyletic Platypodinae.

#### Adenosine deaminase 2 (ADA2)

We amplified and sequenced the *ADA2* gene from 14 species (54%). Failures were most frequent in weevils other than Scolytinae and Platypodinae. The tree topology (Fig 3g) was largely congruent with our predefined clades (A, C, D, E; all pp≥0.95), except Ipini (clade B). The tree was rooted on a monophyletic Platypodinae.

#### RNA-associated protein CTR9 (CTR9)

A single primer pair resulted in the amplification and sequencing of 13 sequences (50%), mainly in Scolytinae, with much lower amplification rates in other weevil subfamilies (1 sequence). The amplified gene fragment revealed a simple structure with a single intron in many species, but was absent in the entire tribe Scolytini and a few other Scolytinae species. The two exons presented a total sequence length of 627 bp (209 amino acids). The phylogeny recovered three pre-defined clades (A, B and D), two of them highly supported (A and D) while resolution at deeper nodes was generally low (Fig 3h).

#### Cyclin-C (CCNC)

A 384 bp fragment (introns excised) was amplified for 20 species (77%), with relatively good taxon coverage among the different groups. The alignment included three long introns which may cause amplification and sequencing problems. The phylogeny based on this marker revealed a monophyletic Platypodinae (pp = 1) that formed the sister group to the advanced weevils (Curculionidae sensu Alonso-Zarazaga and Lyal 1999, pp = 1). All smaller clades were congruent with previous phylogenies, albeit only three clades were strongly supported (A, D and E, pp = 1), whereas the larger group of Scolytinae was paraphyletic with respect to two other weevil species (Fig 3i).

#### Chitin deacetylase 4 (Cda4)

*Cda4* sequences were obtained from a total of 13 beetle species (50%). This marker amplified few weevils other than Scolytinae (2 sequences) and failed to amplify species in the tribe Scolytini. The gene structure was relatively simple with 3 short introns (<100bp), with the first and the third intron present in the majority of the species, while the second one was absent in all Platypodinae and Hylurgini species. The phylogeny based on a 410 bp long coding fragment (136 amino acids) showed monophyly for group A (pp = 1) and E, while Hylurgini (group C) was paraphyletic (Fig 3j).

#### Histone deacetylase Rpd3 (HDAC Rpd3)

*HDAC Rpd3* represents the longest gene fragment selected in this study. This gene was amplified and sequenced for 13 species (50%), with the longest fragments reaching more than 1700 bp due to the presence of introns. A total of 5 introns were present in one species (*Platypus impressus*), while the other species showed a high variability in intron numbers (1–4) with intron 4 particularly long in *Kissophagus hederae* (571 bp). The final alignment, with introns removed, resulted in 858 nucleotide positions coding for 286 amino acids. We did not amplify any species in the tribe Scolytini (clade D) and we had limited success with Ipini (B) and in weevils other than Scolytinae and Platypodinae (Fig 3k). The phylogeny based on these sequences showed a largely unstructured tree, with only clades A and F recovered (pp = 1 and pp = 0.94 respectively), and partially so in Hylurgini (clade C: *Hylastes attenuatus*, *Tomicus piniperda* and *D*. *ponderosae*, pp = 0.98).

#### Arrestin 2 (Arr2)

*Arr2* showed high degree of PCR and sequencing success in Scolytinae and in some other weevils, obtaining a total of 20 sequences (77%). The alignment of our new *Arr2* sequences contained three introns. At the beginning of the second exon, the coding region varied in length due to triplet indels. One example of atypical intron borders was encountered in the first intron (GC-AG), in *Premnobius caevipennis*. Three predefined clades were recovered (A, pp = 1; B, pp = 0.96; E, pp = 1), with two other groups only partly resolved (clade C, pp = 0.98; D, pp = 0.99). The overall tree topology was largely congruent with established phylogenies, where clades A and B were recognized as sister lineages with maximum node support (Fig 3l). The tree was rooted on a monophyletic Platypodinae.

#### Flap endonuclease 1 (FEN1)

*FEN1* sequences were obtained from 15 different species (58%). The alignment of nucleotide sequences revealed three introns that were present in the majority of the species. The coding region was 417 bp long and translated into 139 amino acids. The phylogeny was well resolved and recovered highly supported monophyletic groups corresponding to the clades A, B, C, and D (Fig 3m). In addition, the sister clades A and B were correctly reconstructed (pp = 0.98), and Platypodinae (one species) was, in the absence of other advanced weevils, placed as sister to Scolytinae.

#### Elongation factor 2 (EF2)

We obtained *EF2* sequences from 14 species (54%), but only from species in Scolytinae and Platypodinae. Additional unspecific amplifications of *EF2* were also obtained (7 sequences), mainly from fungi and nematodes. The amplified fragment contained two long introns up to 300 bp, but occasionally longer in a few species (Table 2). Bayesian analysis of 621 aligned nucleotides (207 amino acids) showed a partially correct phylogeny that included several highly supported clades (A, D and E, all with pp = 1). The monophyly of Hylurgini (clade C) was only weakly supported (Fig 3n). The tree was rooted on a monophyletic Platypodinae.

#### Heat shock protein 70 (Hsp70)

Partial *Hsp70* gene was amplified in 14 species (54%) and contained one or two introns. Only the second intron was present in the majority of amplified species. With introns excised, the alignment consisted of 567 nucleotides coding for 189 amino acids. This marker performed particularly well in Hylurgini and Hylesinini (clade C) with 5 out of 6 samples amplified. The phylogeny contained a well resolved clade C (pp = 0.91) and D (Scolytini, pp = 1), while the remaining parts of the tree topology formed largely a polytomy (Fig 3o). Unspecific PCR amplification and sequencing of fungi and nematodes occurred in four samples. Furthermore, paralogous copies, characterized by a triplet insertion in weevils, were identified based on phylogenetic analysis of all available sequences (S3 Fig).

#### Regulator of chromosome condensation 1 (RCC1)

A short fragment consisting of 303 bp (intron excised) was amplified for 13 species (50%). The sequenced gene fragment contained one intron in all species, except *Hylesinus varius*, and the exons could be translated into 101 amino acids. The primers showed very low success in weevils other than Scolytinae, amplifying only two species in group E (Platypodinae) and one species of Cossoninae. The primers did not amplify this gene in the tribe Scolytini (D). Occasional unspecific amplifications were observed (4 sequences, from fungi and nematodes). The phylogeny based on this marker was mainly congruent with established relationships and showed no evidence of multiple copies (Fig 3p). Platypodinae (E, pp = 1), Dryocoetini (A, pp = 0.91) and a subclade of Hylurgini (C, pp = 1) were recovered.

### Phylogenetic signal

**Fig 4:**
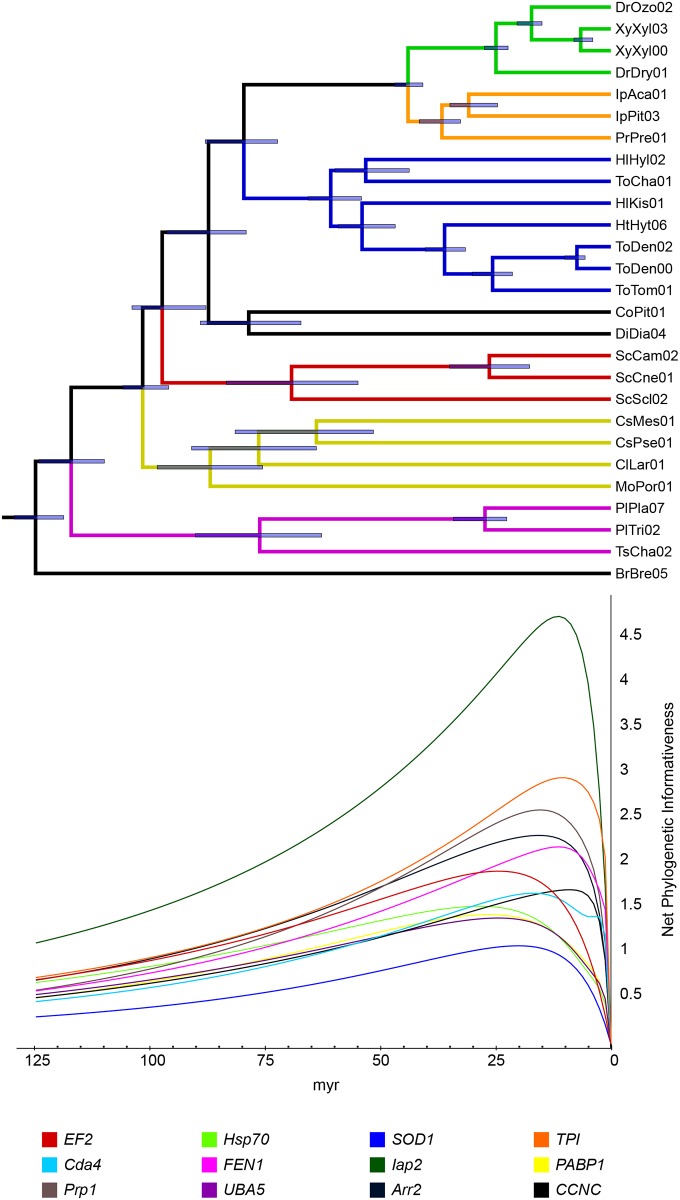
Phylogentic informativeness profiles. The K2P model was used to estimate substitution rates in HyPhy as implemented in the software PhyDesign. Different evolutionary models produced similar results (data not shown). The dated phylogenetic tree was obtained using BEAST v1.8.2.

Phylogenetic informativeness (PI) profiles varied considerably between the selected markers, showing different degrees of signal across the more than 100 Ma of weevil evolutionary history (Fig 4). The net PI values showed a marked decline for all markers towards the Cretaceous era. *Iap2* displayed the highest PI peak in recent times, followed by four other markers with lower PI profiles (*TPI*, *Prp1* and *Arr2*, *FEN1*). The gene *EF2* showed a diverse profile, having lower PI for recent times but relatively more PI than *FEN1* and *Arr2* at more ancient times. *PABP1*, which presented the highest homoplasy level among the selected genes (S3 Table), showed an intermediate PI profile, following the same trend of *Hsp70*, *Cda4*, *CCNC* and almost identical to *UBA5*. *Cda4* and *CCNC* showed higher PI in recent times while *Hsp70* maintained marginally higher PI for ancient times. The gene with the lowest PI value was *SOD1*. Four markers (*HDAC Rpd3*, *ADA2*, *RCC1* and *CTR9*) were not included in the analysis due to missing data.

### Additional genes for lower level phylogenetics

**Fig 5:**
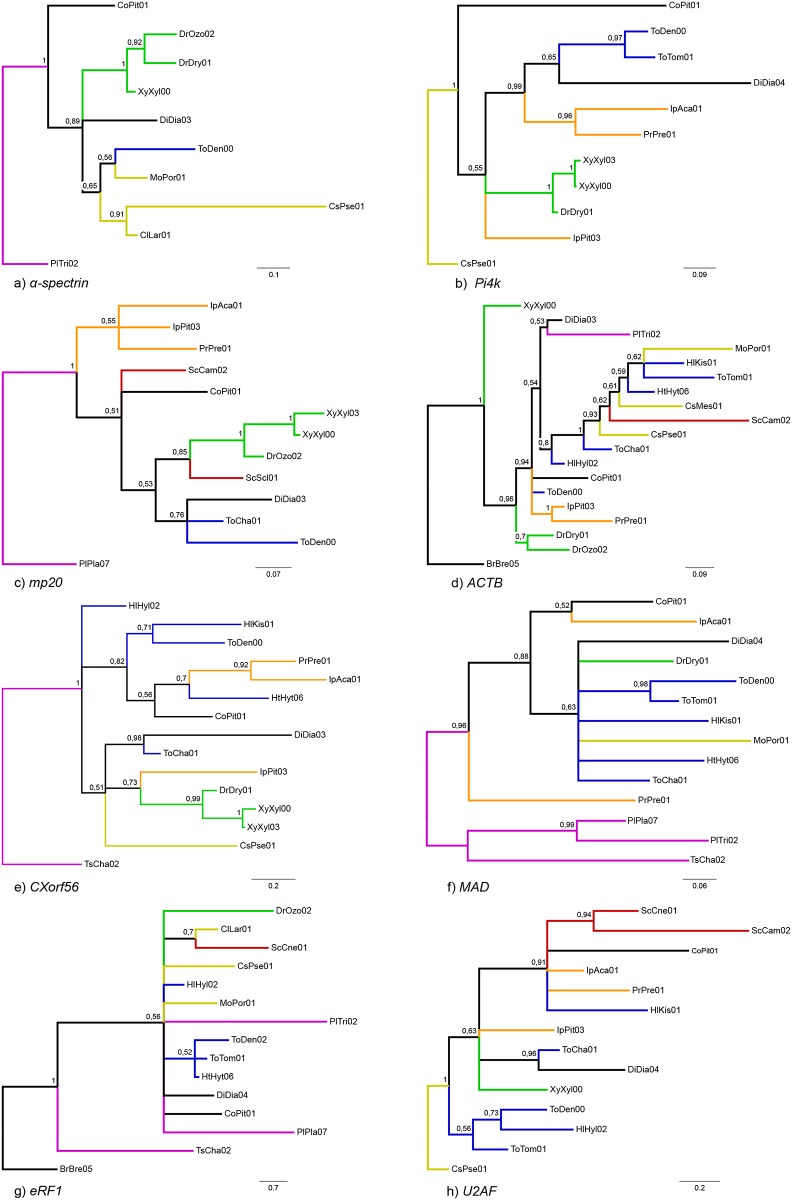
Phylogenetic trees resulting from Bayesian analyses of 8 excluded gene fragments.

One of the main characteristics shared by several of the 18 genes that were not selected was the generally low, and sometimes clade-specific, PCR and sequencing success (S3 Table). These genes also exhibited many problems in phylogeny reconstruction when sufficient data were obtained, including failure to recover well-established clades (Fig 5). For example, very few sequences were acquired for *α-spectrin*, with no sequences obtained for three of the groups (B, C and D), producing a tree topology with only one correct clade recovered (A, pp = 1) and therefore difficult to evaluate (Fig 5a). A similar situation was reported for *phosphatidylinositol 4-kinase type 2-alpha* (*Pi4k*) where no sequences were obtained for the clades D and E, but two clades (A, pp = 1 and C, pp = 0.97) were recovered correctly (Fig 5b), and a third group was nearly monophyletic (B, excluding *Pityogenes quadridens*, pp = 0.96). For *muscular protein 20* (*mp20*) we obtained a higher number of sequences (12), with two monophyletic groups recovered (clade A, pp = 1 and B, pp<0.95), but with group D (Scolytini) not monophyletic (Fig 5c). In the case of the *beta-actin* gene (*ACTB*), sequences were obtained from 18 different species, including 5 species of Hylurgini. However, the phylogeny recovered only one of the youngest clades (B, pp = 1), while all other groups were largely paraphyletic (Fig 5d). In the *chromosome X open reading frame 56* gene (*CXorf56*), only the youngest group (clade A, pp = 0.99) was correctly recovered (Fig 5e) whereas closely related species did not group together. Another poorly performing gene was *MAD*, with a phylogenetic tree showing a large polytomy that included a highly paraphyletic Hylurgini (clade C). This gene nevertheless distinguished Platypodinae (pp = 0.96) from all other advanced weevils at the root of the tree (Fig 5f). A similar situation was also observed for the *eukaryotic peptide chain release factor subunit 1* (*eRF1*) gene. The phylogeny largely formed a polytomy (Fig 5g), and included many paraphyletic groups, including Platypodinae (clade E). The phylogeny for *splicing factor U2F* showed a largely unstructured tree with generally low support (Fig 5h), with only Scolytini monophyletic (clade D, pp<0.95).

The remaining 10 of the 18 genes with shallow level phylogenetic utility generally exhibited low PCR and sequencing success (5–9 sequences), and showed clade-specific amplification (see S3 Table). A correct tree topology was recovered for *dihydrolipoamide dehydrogenase E3* (*dldE3*) which showed a congruent and well supported phylogeny for three clades (A, B and C, all with pp>0.95) and also recovered a node including A+B (pp = 0.99). The low number of sequences obtained (7) was the main reason to exclude this gene. *Alanyl-tRNA synthetase* (*AATS*), *F-box only protein 11* and *Na+/K+ ATPase alpha subunit* (*NaK*) displayed very low PCR and sequencing success. The first of these recovered clade A (pp = 1), the second clade A and B (pp = 1 and pp = 0.99 respectively) while the third one did not produce enough sequences to enable hypotheses testing. *Hsp90* revealed amplification of eight species in Scolytinae, but not other weevils. The phylogeny was consistent with clade A (pp = 0.97) and partially so for clade C (3 species pp<0.95). The alignment of *Hsp90* revealed no intron but the coding region presented variable length due to the presence of indels. Primers for the two genes *mannose-1-phosphate guanyltransferase α C1-tetrahydrofolate synthase* (*C1-THF*) and *uracil-DNA degrading factor* amplified well in Hylurgini. Finally, *gelsolin* and *elongation initiation factor 5C* (also known as *krasavietz - 5MP*) revealed unstructured tree topologies. The first gene recovered only clade D (pp = 1) while the second supported clade E (pp = 1) and in part clade C. Additional information on suggested subfamily/tribe/genus specific markers were reported in supplementary material (S3 Table).

## Discussion

Phylogenetic studies on insects have generally suffered from a lack of coordination in establishing a common set of nuclear markers [73]. Most efforts were invested in butterflies and bees [31, 37], with other related groups occasionally taking advantage of such developments [74]. Beetles are one of the many groups lagging behind in terms of phylogenetic marker availability. With the presentation of 16 protein coding genes, which are here shown to be informative in weevil phylogenetics, and the suggestion of 18 additional, but less developed, genes as potential phylogenetic markers at various taxonomic levels, we have at least partly remedied this situation. Indeed, many of the 16 best markers were relatively easy to amplify with one or two primer pairs, with a PCR success ratio between 50 and 100%. Direct sequencing was facilitated by the high proportion of single bands produced in the PCR of these genes. Only occasional events of unspecific amplification occurred and most sequences could be aligned unambiguously and translated into amino acids.

Further optimization of primers is required to enable amplification across a broader range of weevils and other beetle groups. This is particularly relevant to the many unsuccessful genes that we screened, which may amplify with a better design of primers. In such a brief screening of candidate genes it is likely that promising markers were overlooked. The gene *α-spectrin*, as one example, may deserve further attention as one of very few genes previously screened for beetles [23]. Unfortunately, the primers designed in this study amplified mainly Xyleborini and Dryocoetini, but not the majority of other tribes. We also continued our previous screening of the *NaK* gene [9], which again was particularly positive for Ipini, Dryocoetini and Xyleborini, with potential application at lower level phylogeny.

Only one marker amplified in all samples (*PABP1*). This gene, and three additional ones (*TPI*, *UBA5* and *Prp1*) with comparable high amplification rates, shared a pattern of simple intron structure, which may facilitate the amplification process. Other genes could be almost as easily amplified (*Arr2*, *Iap2*, *CCNC*), but required more efforts in the alignment procedure due to the presence of highly variable regions and/or introns. For all the other genes, improved primer design seems required to obtain PCR and sequencing regularity at appreciable levels such as in nymphalid butterflies [32] or dolichoderine ants [39]. Suboptimal primer design was most evident in cases where failures in amplification were taxon-specific, for instance *TPI*, *HDAC Rpd3*, *Cda4* and *RCC1* in species of the tribe Scolytini. Other genes such as *ADA2*, *Hsp70*, *FEN1* and *CTR9* were amplifying Scolytinae, which was our main target group, but failed in most other weevils.

Degenerate primers tend to amplify non-targeted regions for several of the screened genes. However, only two genes with short amplified fragments (*SOD1* and *RCC1*) were regularly affected by this kind of problem, and occurred less frequently in *CXorf56*, *Hsp90* and *eRF1*. The amplification of other gene copies is a relatively common problem in PCR based methods and at least three routinely used markers (*COI*, *EF-1α*, *enolase*) in bark and ambrosia beetles are occasionally burdened with such complexity [10, 68, 75]. In other cases, such as *EF2* and *Hsp70*, the same gene copy was unintentionally amplified from other beetle-associated organisms (fungi and nematodes), probably due to the conserved nature of these genes [68]. When we tested nuclear markers for orthology assessment in arthropods (OrthoDB v9), *Hsp70* was one of the few genes which resulted present in multiple copies in the large majority of the species in the database (98%). In our study, the presence of *Hsp70* paralogs was clearly demonstrated based on BLAST search, strongly deviating amino acid substitution patterns and long phylogenetic branches of paraphyletic groups (S4 Fig). Although three other genes (*Iap2*, *Arr2* and *SOD1*) are rarely in single copy in the arthropod genomes, our study did not provide any clear evidence of paralogy in beetles.

Two markers (*HDAC Rpd3* and *CCNC*) were particularly problematic due to the many long introns they contained (up to 5 in *HDAC Rpd3*) and they require internal primers for more effective amplification and sequencing. The presence of long and/or numerous introns seems widespread in beetles. This insect order has generally a higher number of introns compared to other insects [76], particularly so in the phytophagan beetles [23]. For example, a 300 bp short fragment of the gene *Wingless*, which is widely used in insect phylogeny, contains three complicated introns in weevils, but it is intron free in adephagan beetles and most other insect orders. On the other side are weevil sequences of *TPI* simpler than those of coccoidean Hemiptera which have two extra introns and one hypervariable indels region [77]. Only two introns were present in the majority of weevils, although highly variable in Hylurgini and four additional species. Similar situations, with lack of conserved intron patterns within clades, were observed for genes such as *CTR9*, *HDAC Rpd3* and *Cda4*, contrasting the long held argument that intron structure is a conserved and therefore useful phylogenetic marker [78, 79].

A further complicating feature in the alignments of *Arr2* and *Iap2* involved variable coding regions that contained different numbers of triplet nucleotide indels. Because indel-rich regions are difficult to align, they could potentially introduce unwarranted noise in the phylogenetic signal. However, the removal of these ambiguous regions did not affect tree topologies resulting from independent analyses of each of these genes. Indel-rich regions of *Arr2* occur in species from other insect orders (BLAST analyses), which further document natural and widespread variation in this trait. *Iap2* is much less known in terms of indels variation and our data were only comparable to other GenBank sequences in the second more conserved exon.

The process of evaluating and ranking different markers in terms of phylogenetic utility is a complex task. Rates are not always inversely correlated with phylogenetic resolution and clade support [80] and only the implementation in large taxonomic samples represents the ultimate test of a phylogenetic marker performance. Our gene classification based on phylogenetic utility that was assessed according to clade congruence and phylogenetic informativeness (PI) must therefore be taken as a preliminary proxy for a marker’s phylogenetic signal [81, 82, 83]. It will be particularly interesting to observe the contribution of *Iap2* in a larger data set given its much higher PI compared with other markers. *Iap2* is a fast evolving gene which, likewise *TPI*, *Prp1*, *FEN1* and *Arr2*, showed a high peak for the Miocene epoch, but it differs from the other genes by maintaining a stronger phylogenetic signal over time. Even though this marker has two variable regions that could have biased the PI profile estimate, the average level of homoplasy was also the lowest for this gene. On the other hand, the tree topology resulting from the phylogenetic analyses was not particularly congruent with previously established relationships.

Only one gene (*FEN1*) produced a tree topology that was largely congruent with all predefined clades, and only three genes (*PABP1*, *FEN1*, *Arr2*) were congruent with the most recent split—between Ipini and Dryocoetini/Xyleborini (Paleocene age)—indicating high substitution rates for most genes in our screening. However, a perfect match between a gene tree and the species tree is rarely observed [84]. Dense taxon sampling and simultaneous analyses of many genes will usually overcome such limitations, building on the hidden support from many genes not visible in single gene analyses [85, 86].

Large amounts of data are usually required to obtain resolution between more ancient groups such as insect orders and families. It is therefore a possibility that 15–20 markers are not sufficient to resolve the weevil phylogeny, including relationships among bark and ambrosia beetles. Data volume is by itself useful as demonstrated by studies on the complete mitochondrial genome of weevils that resolve certain parts of the tree topology [6, 87]. Limiting mitochondrial data to a handful of genes illustrates this point well as resolution fades rapidly [8]. Larger data volumes are now available from nuclear genome sequencing, either in terms of entire genomes [88–90], or transcribed genomes [91, 92]. Each of these approaches has their own disadvantages with respect to high cost and labor intensity. Transcriptome data are furthermore burdened with highly biased gene expressions, for instance the overexpression of ribosomal proteins in ESTs of beetles [93]. A targeted PCR-based approach to sequencing has on these grounds been recommended in phylogenetic analyses [94].

New NGS technologies have lately enabled more specific amplification of conserved sequence regions, bypassing complete genomic or transcriptomic assembly, and thereby reducing the dataset to a core of comparable informative sequences which are more suitable for phylogenetics [95, 96]. Sequence capture of ultra-conserved elements (UCEs) has enabled high sequence homology [51, 97, 98] and hence, these results are more directly comparable to PCR based sequences. UCEs have a phylogenetic information potential comparable to protein coding genes at the per nucleotide level; however, the large volume of data involving hundreds of loci and more than 100,000 nucleotides provide better resolution and higher support at deep phylogenetic level [99, 100].

It is increasingly being argued that PCR-based methods are becoming redundant in the age of NGS, but this is largely an overstatement. Most sequencing, in fact, occurs at a routine basis, as a tool in integrative taxonomy where a handful of sequences from established markers are sufficient to place a new species in the tree of life. Most laboratories in the world are not yet rigged for the latest NGS in terms of equipment, labor and budgetary concerns. As long as the monthly turnaround rate involves less than 10 genes and 100 taxa, the time and cost doing traditional PCR and sequencing is much lower [100]. Recognizing that small data sets are not only less expensive, but also can be sufficiently informative, the reliance on PCR and Sanger sequencing will continue as the best option for many small scale studies also in the future. In fact, modest data sets of a few thousands of nucleotides (5–10 genes) can be almost as informative as large collections of UCEs [100, 101]. With approximately 80–90% congruence in topology, one may reconsider if sequencing of UCEs is always the best option despite the generally higher node support obtained for this type of data.

## Conclusion

This study has revealed the many difficulties in selecting and optimizing new markers for weevil phylogenetics. Other beetle groups may be less problematic than weevils [23], but beetles in general are much more challenging in this respect as compared to Hymenoptera and Lepidoptera [32, 36, 86, 102]. Nevertheless, this study provides a step forward in PCR-based sequencing of beetles and we hope that these new markers will provide a useful toolbox for beetle phylogenetics, particularly in studies on more recent divergences where a limited amount of genetic data can enable accurate inference of past evolutionary events.

## Supporting Information

S1 Fig Phylogenetic tree based on Bayesian analyses of 16 concatenated genes both for nucleotides and amino acids. Posterior probability values are reported below the node for the nucleotides analysis (8109 bp), while the pp values above the node refer to the amino acids analysis (2702 aa).
(TIF)

S2 Fig Phylogentic tree based on parsimony analyses of 16 concatenated genes both for nucleotides and amino acids. Bootstrap support values are reported below the node for the nucleotides analysis (8109 bp), while the values above the node indicate the bootstrap support for amino acids analysis (2702 aa).
(TIF)

S3 Fig Average genetic variation for each marker. *p*-distance values for each position and for each gene were calculated across the entire sample, excluding Brentidae to avoid missing data.
(TIF)

S4 Fig Phylogenetic tree based on a fragment of the gene *Hsp70*. Results of Bayesian analysis based on *Hsp70* sequences of weevils and Scolytinae; three different copies of *D*. *ponderosae Hsp70* were included in order to test for paralogs. Six more species were also included in the analysis (CuSib01 = *Sibinia* sp. CgAph02 = *Aphanarthrum capense*, MiLan01 = *Lanurgus xylographus*, MoAmo01 = *Amorphocerus rufipes*, DrCyr02 = *Acanthotomicus* sp. and TsCen01 = *Cenocephalus* sp.). Three different *Hsp70* groups were identified. One group consisted of paralogous copies of *Hsp70* (A), plus two clusters of sequences from fungi (B) and nematodes (C).
(TIF)

S1 File XML file used for analyses in BEAST v1.8.2. The file was generated using BEAUTI v 1.8.2.
(XML)

S2 File Additional information on 16 PCR amplified and sequenced genes. (DOCX)

S1 Table GenBank accession numbers for each of the 16 selected genes sequenced in this study. (DOCX)

S2 Table Estimates of evolutionary divergence (*p*-distance) between sequences. For each of the 16 genes, the proportion of different nucleotide sites between sequences was calculated. The most frequently PCR amplified species (*Xyleborus affinis*) was compared with members of the other tribes and subfamilies and the lower value was reported. PIC = Parsimony informative characters, HI = Homoplasy index and RI = Retention index.
(DOCX)

S3 Table Information on markers not developed for higher level phylogenetics. The main problems for further development are reported, together with data on fragment length, and number and length of introns for 18 of these markers. The same information could not be derived for markers with low number of sequences.
(DOCX)
