# Supplementary figures and images for: Genomic Mining of Phylogenetically Informative Nuclear Markers in Bark and Ambrosia Beetles
Source: PLoS One. 2016 Sep 26;11(9):e0163529. doi: 10.1371/journal.pone.0163529 (PMC5036811; doi:10.1371/journal.pone.0163529)

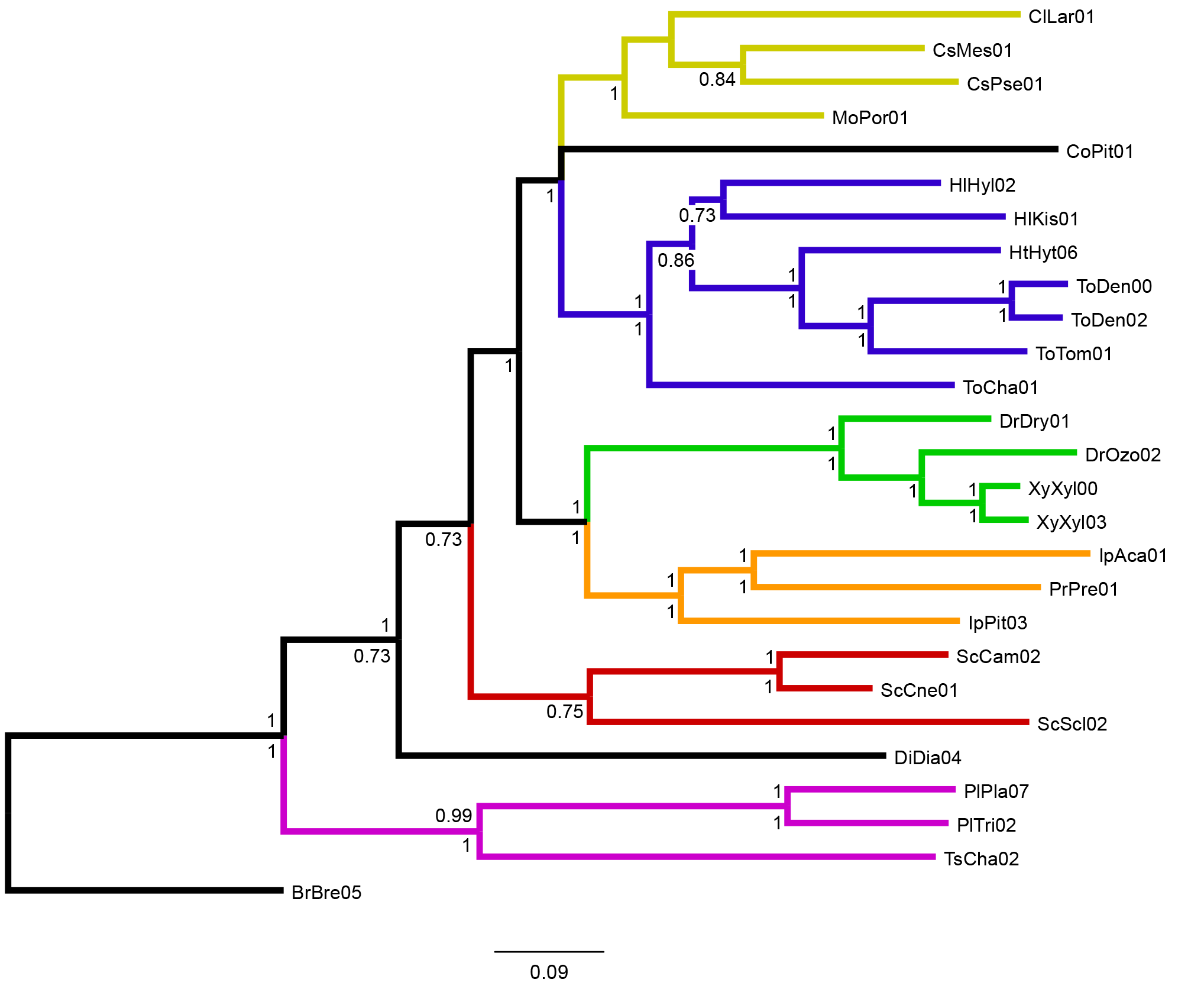

Supplement: S1 Fig — Posterior probability values are reported below the node for the nucleotides analysis (8109 bp), while the pp values above the node refer to the amino acids analysis (2702 aa). (TIF) [file pone.0163529.s001.tif]

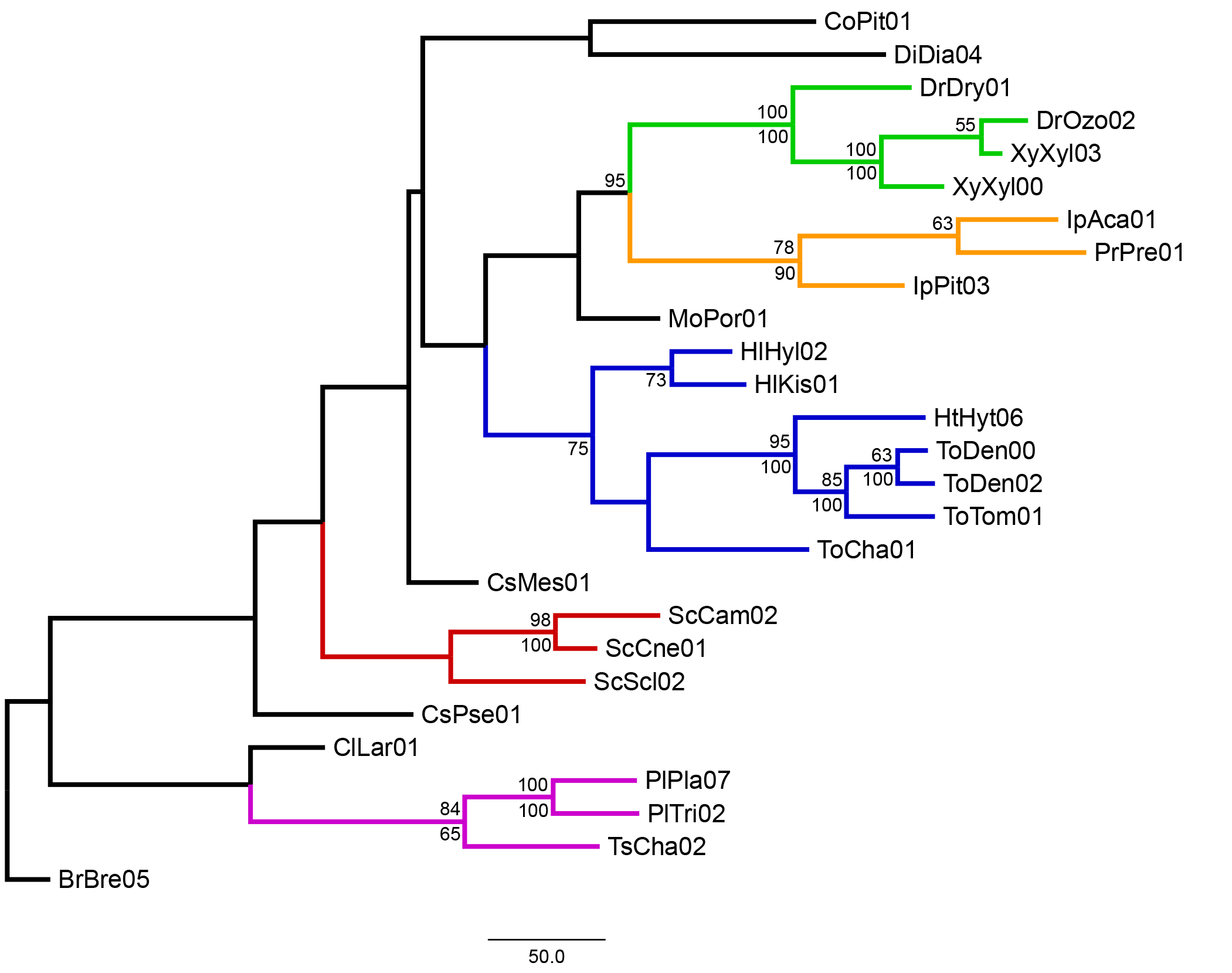

Supplement: S2 Fig — Bootstrap support values are reported below the node for the nucleotides analysis (8109 bp), while the values above the node indicate the bootstrap support for amino acids analysis (2702 aa). (TIF) [file pone.0163529.s002.tif]

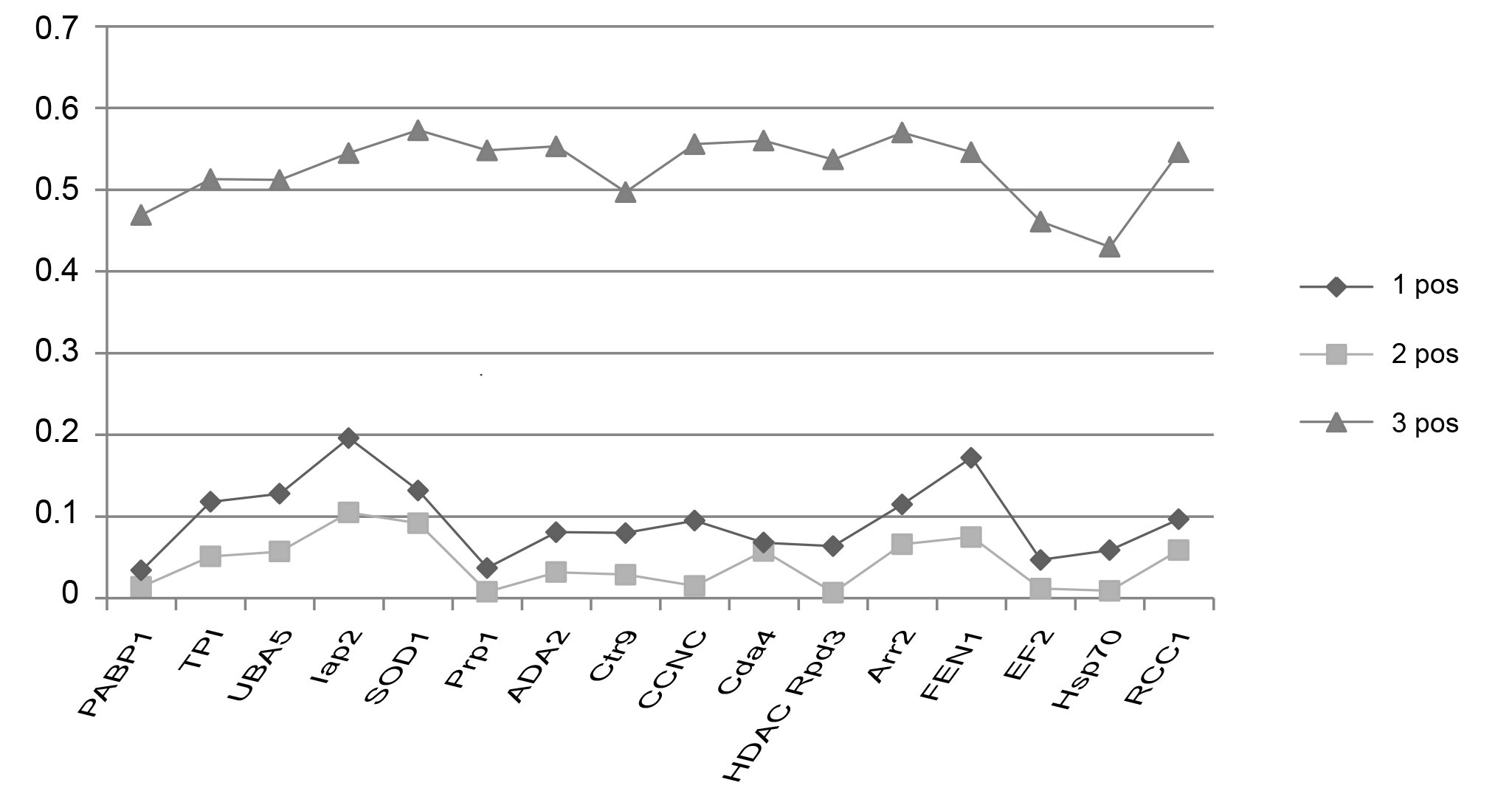

Supplement: S3 Fig — p-distance values for each position and for each gene were calculated across the entire sample, excluding Brentidae to avoid missing data. (TIF) [file pone.0163529.s003.tif]

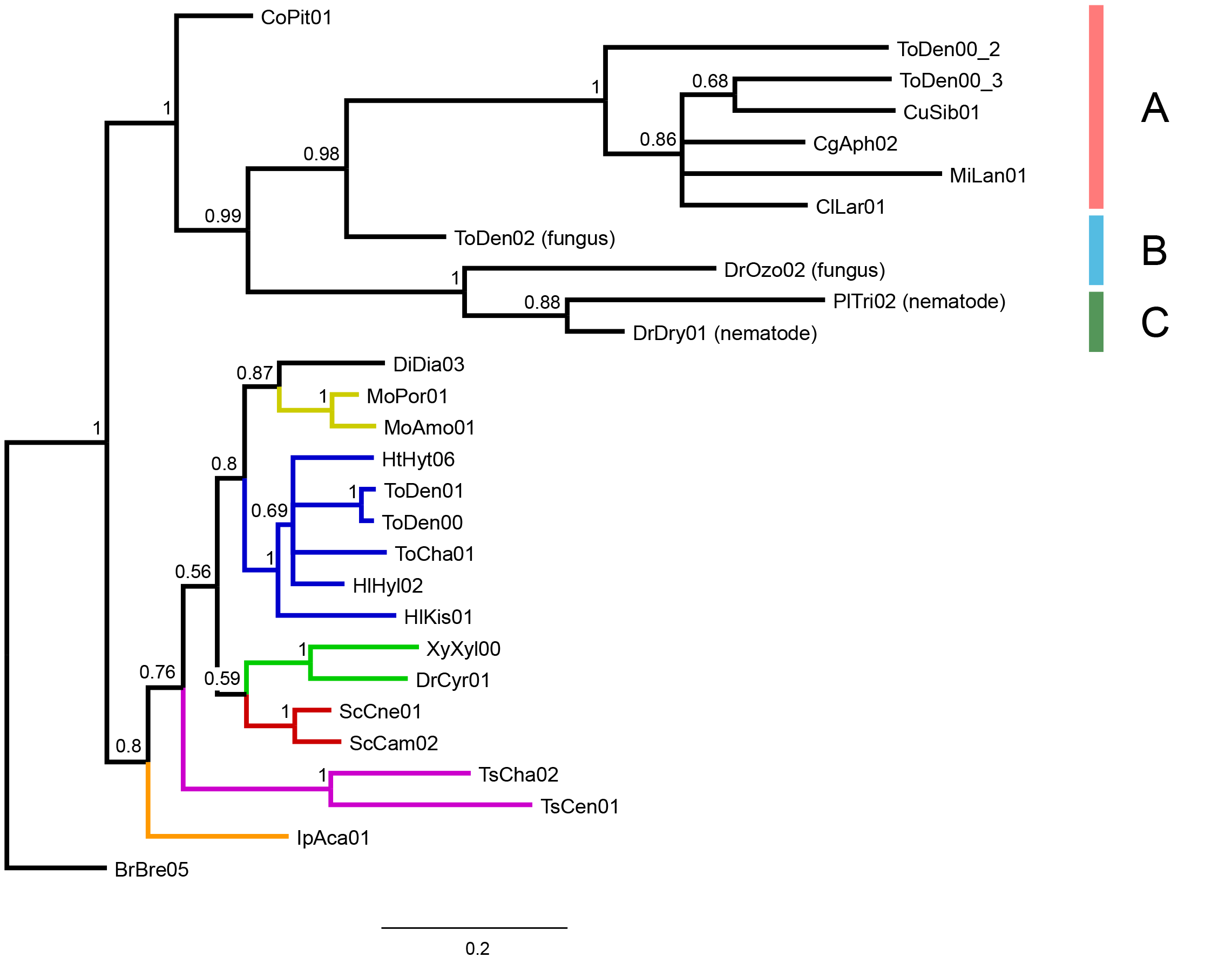

Supplement: S4 Fig — Results of Bayesian analysis based on Hsp70 sequences of weevils and Scolytinae; three different copies of D. ponderosae Hsp70 were included in order to test for paralogs. Six more species were also included in the analysis (CuSib01 = Sibinia sp. CgAph02 = Aphanarthrum capense, MiLan01 = Lanurgus xylographus, MoAmo01 = Amorphocerus rufipes, DrCyr02 = Acanthotomicus sp. and TsCen01 = Cenocephalus sp.). Three different Hsp70 groups were identified. One group consisted of paralogous copies of Hsp70 (A), plus two clusters of sequences from fungi (B) and nematodes (C). (TIF) [file pone.0163529.s004.tif]
