# Supplementary material for: Genomic Mining of Phylogenetically Informative Nuclear Markers in Bark and Ambrosia Beetles
Source: PLoS One. 2016 Sep 26;11(9):e0163529. doi: 10.1371/journal.pone.0163529 (PMC5036811; doi:10.1371/journal.pone.0163529)
Supplement: S2 File — (DOCX) [file pone.0163529.s006.docx]

**Gene description**

***Polyadenylate binding protein 1* (*PABP1*)**

*PABP1* protein interacts with the mRNA 3’-poly(A) tail in eukaryotes and together with the poly(A) tail it is involved in maintenance of the mRNA stability and correct translation [1]. In insects, *PABP1* was extensively characterized in *Drosophila melanogaster* [2, 3]. So far, *PABP1* has not been used in insect phylogenetics, but was previously used in the reconstruction of vertebrate phylogeny [4].

Alternative reverse primer.

PABP1_revB: CGCCCRTTCATYTCWGTMACSGC

***Triose-phosphate isomerase* (*TPI*)**

*TPI* is a key enzyme in the glycolysis pathway that catalyzes the reversible interconversion of dihydroxyacetone phosphate and D-glyceraldehyde 3-phosphate [5]. This gene was reported to be present in single copy in *Drosophila melanogaster* [6]. The *TPI* gene has been sporadically used to investigate the phylogeny of various insect groups and it is emerging as a valuable nuclear marker [7-10].

***Ubiquitin-like modifier activating enzyme 5* (*UBA5*)**

*UBA5* is a conserved gene coding for a protein which plays an essential role in protein binding processes and in the activation of the ubiquitin-fold modifier 1 (Umf1) by forming a high-energy thioester bond [11].

***Inhibitor of apoptosis 2* (*Iap2*)**

*IAP* proteins constitute a large family of structurally and functionally related proteins which are able to bind and inhibit caspases, a class of cysteine proteases involved in propagating apoptotic signals within the cell [12]. The Baculovirus IAP repeat [13] is a characteristic domain of *IAPs* which is present in one or more copies and shows zinc-finger like motives. The BIR domain is necessary for protein interaction with a number of apoptotic initiating factors, including invertebrate death inducers. In insects, *Iap2* is also involved in different signaling pathways and in innate immune response to Gram-negative bacteria infection [14].

***Cu-Zn superoxide dismutase 1* (*SOD1*)**

Superoxide dismutases are important cellular enzymatic defenses against the detrimental reactive oxygen species generated by aerobic metabolism. These ubiquitous metal enzymes catalyze the dismutation of the superoxide anion (O_2_^−^) to hydrogen peroxide H_2_O_2_, which is converted into H_2_O by catalase and peroxidases [15]. In mammals, at least three distinct *SODs* with similar enzymatic function have been identified and characterized, differing in the metal cofactors used and cellular compartmentalization [16] while the amount of information on these enzymes in insects is limited. The *SOD1* gene was extensively studied in some species of Diptera and used in phylogeny reconstruction of *Drosophila* and related genera [17].

***Pre-mRNA-splicing factor ATP-dependent RNA helicase PRP1* (*Prp1*)**

Pre-mRNA-splicing factors denote a large number of different enzymes involved in the assembly and disassembly, activation, catalysis and recycling of the spliceosomes [18]. The *Prp1* gene is poorly investigated in insects and details on the function and specific interaction with other spliceosomal components are lacking.

***Adenosine deaminase 2* (*ADA2*)**

Adenosine deaminases (*ADA*s) are primarily involved in the deamination of adenosine and deoxyadenosine into inosine nucleosides, protecting the cells against the accumulation of toxic levels [19]. Genome sequencing has shown that these genes are present in multiple copies in different Metazoa taxa. Limited information is available on these proteins and their role in insects.

***RNA-associated protein CTR9* (*CTR9*)**

The protein *CTR9* is a component of the Paf1 complex (Pafc), an RNA polymerase II associated factor which control several steps in the transcription process [20]. Data on the specific role and interaction of the *CTR9* protein with other components of Pafc are not available for insects. This gene has not previously been used in insect phylogenetics.

***Cyclin-C (CCNC)***

Cyclins are a group of proteins responsible for controlling the progression of the cell cycle by activating cyclin-dependent kinase (CDK) enzymes [21]. *Cyclin-C* was shown to interact with the CDK8, triggering the phosphorylation of the carboxy-terminal domain of the large subunit of RNA polymerase II and regulating its function [22]. The CCNC gene is poorly investigated in insects, with the exception of *D. melanogaster* [22] and it has never been used in beetle phylogenetics.

***Chitin deacetylase 4* (*Cda4*)**

Chitin deacetylases (*CDAs*) form a large family of metalloproteins involved in extracellular chitin modification. These enzymes catalyze the deacetylation of chitin through the hydrolysis of N-acetamido bonds to form chitosan [23, 24]. *CDAs* were characterized in marine bacteria, fungi and insects, where the number of gene copies varies from five to nine [25].

***Histone deacetylase Rpd3* (*HDAC Rpd3*)**

Histone deacetylases are a class of enzymes belonging to a large superfamily of genes that regulate the activity of histones, removing acetyl groups (O=C-CH_3_) from the aminon acid lysine, and controlling the DNA-histone mediated packing mechanism [26]. Epigenetic gene expression is regulated by acetylation and de-acetylation processes, therefore these enzymes play a crucial role in cell activity. Besides, histone deacetylation mechanism is involved in transcriptional regulation, cell cycle progression, DNA damage response, osmotic stress response and other cell developmental events.

***Arrestin 2* (*Arr2*)**

*Arr2* is a member of the non-visual arrestin group and it is mainly localized in the nucleus [27]. *Arr2* is involved in the regulation of histone acetylation and gene transcription. Environmental signals and chemicals are perceived through binding to phosphorylated active G-protein-coupled receptors. So far, no arrestin or arrestin-like proteins have been found in plants or fungi. This protein has been phylogenetically characterized in *Maruca vitrata* (Lepidoptera: Crambidae) and other holometabolous insects [28]. The genomic DNA sequence in *M. vitrata* presents a total length of 1779 bp and consists of 6 exons. A relatively high expression of mRNA in the late pupal stages was reported, suggesting multiple developmental functions. The alignment of amino acid sequences with those of other insects revealed highest similarity (98.8 %) to the monarch butterfly, and much lower similarity (53.9–60.7 %) to putative homologs in Hymenoptera and Diptera.

***Flap endonuclease 1* (*FEN1*)**

Endonucleases are enzymes involved in cleaving the phosphodiester bond within a polynucleotide chain. The *FEN1* gene is involved in DNA replication and repair and considered a central component of cellular DNA metabolism since its efficiency and specificity is critical in maintaining genome fidelity. Besides, this gene has been shown to be involved in apoptotic process [29]. *FEN1* is an ancient protein that is conserved over evolutionary time to coordinate many essential DNA transactions [30].

Alternative primers.

FEN1_forA: GMAARGTGGCMATYGAYGC

FEN1_revB: GCGTCCATRTCYTCMGTKGC

***Elongation factor 2* (*EF2*)**

Eukaryotic elongation factors (*eEFs*) are enzymes involved in cellular protein synthesis, controlling translational mRNA processes in the ribosome. *EF2* is involved in the translocation of the peptidyl-tRNA from the A-site to the P-site of the ribosome, thus liberating the A-site for the following aminoacyl-tRNA [31]. Only *EF-1α* has been extensively characterized in several insect orders, especially in terms of copy number and intron structure [32, 33]. *EF2* is rarely used in phylogenetics of invertebrates, including several Lepidoptera studies, however mainly based on mRNA sequences [34].

***Heat shock protein 70* (*Hsp70*)**

The highly conserved and ubiquitously expressed genes coding for heat shock proteins constitute a large and complex family. These proteins act as molecular chaperones with an important role in correct protein folding and cell protection from stress [35]. Several distinct *Hsp70* paralogs exist in eukaryotes, often localized in different intracellular compartments and multiple copies may therefore also be present in insects.

***Regulator of chromosome condensation 1* (*RCC1*)**

*RCC1* plays important roles in the regulation of gene expression in the eukaryotic cell cycle [36]. This gene encodes a protein which binds to chromatin and interacts with *ran* (a nuclear GTP-binding protein), acting as a guanine-nucleotide dissociation stimulator that promote the loss of bound GDP and the uptake of fresh GTP. However, this gene remains poorly characterized in insects.

1. Gorlach M, Burd CG, Dreyfuss G. The mRNA poly(A)-binding protein: localization, abundance, and RNA-binding specificity. Exp Cell Res. 1994; 211(2): 400-407. Epub 1994/04/01. doi: 10.1006/excr.1994.1104. PubMed PMID: 7908267.

2. Lefrere V, Vincent A, Amalric F. *Drosophila melanogaster* poly(A)-binding protein: cDNA cloning reveals an unusually long 3'-untranslated region of the mRNA, also present in other eukaryotic species [corrected]. Gene. 1990; 96(2): 219-225. Epub 1990/12/15. PubMed PMID: 2125288.

3. Roy G, Miron M, Khaleghpour K, Lasko P, Sonenberg N. The *Drosophila* poly(A) binding protein-interacting protein, dPaip2, is a novel effector of cell growth. Mol Cell Biol. 2004; 24(3): 1143-1154. Epub 2004/01/20. PubMed PMID: 14729960; PubMed Central PMCID: PMCPmc321445.

4. Fong JJ, Fujita MK. Evaluating phylogenetic informativeness and data-type usage for new protein-coding genes across Vertebrata. Molecular phylogenetics and evolution. 2011; 61(2): 300-307. Epub 2011/07/12. doi: 10.1016/j.ympev.2011.06.016. PubMed PMID: 21742044.

5. Hasson E, Wang IN, Zeng LW, Kreitman M, Eanes WF. Nucleotide variation in the triosephosphate isomerase (Tpi) locus of *Drosophila melanogaster* and *Drosophila simulans*. Molecular biology and evolution. 1998; 15(6): 756-769. Epub 1998/06/06. PubMed PMID: 9615457.

6. Shaw-Lee RL, Lissemore JL, Sullivan DT. Structure and expression of the triose phosphate isomerase (Tpi) gene of *Drosophila melanogaster*. Mol Gen Genet. 1991; 230(1-2): 225-229. Epub 1991/11/01. PubMed PMID: 1720860.

7. Tachi T. Molecular phylogeny and host use evolution of the genus *Exorista* Meigen (Diptera: Tachinidae). Molecular phylogenetics and evolution. 2013; 66(1): 401-411. Epub 2012/11/06. doi: 10.1016/j.ympev.2012.10.017. PubMed PMID: 23123315.

8. Salazar C, Jiggins CD, Taylor JE, Kronforst MR, Linares M. Gene flow and the genealogical history of *Heliconius heurippa*. BMC Evol Biol. 2008; 8: 132. Epub 2008/05/06. doi: 10.1186/1471-2148-8-132. PubMed PMID: 18454858; PubMed Central PMCID: PMCPmc2391162.

9. Hardy NB. Phylogenetic utility of dynamin and triose phosphate isomerase. Systematic Entomology. 2007; 32(2): 396-403. doi: 10.1111/j.1365-3113.2007.00377.x.

10. Sohn J-C, Regier JC, Mitter C, Adamski D, Landry J-F, HeikkilÄ M, et al. Phylogeny and feeding trait evolution of the mega-diverse Gelechioidea (Lepidoptera: Obtectomera): new insight from 19 nuclear genes. Systematic Entomology. 2016; 41(1): 112-132. doi: 10.1111/syen.12143.

11. Komatsu M, Chiba T, Tatsumi K, Iemura S, Tanida I, Okazaki N, et al. A novel protein-conjugating system for Ufm1, a ubiquitin-fold modifier. Embo j. 2004; 23(9): 1977-1986. Epub 2004/04/09. doi: 10.1038/sj.emboj.7600205. PubMed PMID: 15071506; PubMed Central PMCID: PMCPmc404325.

12. Kocab AJ, Duckett CS. Inhibitor of apoptosis proteins as intracellular signaling intermediates. Febs j. 2015. Epub 2015/10/16. doi: 10.1111/febs.13554. PubMed PMID: 26462035.

13. Keeling CI, Yuen MM, Liao NY, Docking TR, Chan SK, Taylor GA, et al. Draft genome of the mountain pine beetle, Dendroctonus ponderosae Hopkins, a major forest pest. Genome Biol. 2013; 14(3): R27. Epub 2013/03/30. doi: 10.1186/gb-2013-14-3-r27. PubMed PMID: 23537049; PubMed Central PMCID: PMCPmc4053930.

14. Leulier F, Lhocine N, Lemaitre B, Meier P. The *Drosophila* inhibitor of apoptosis protein DIAP2 functions in innate immunity and is essential to resist gram-negative bacterial infection. Mol Cell Biol. 2006; 26(21): 7821-7831. Epub 2006/08/09. doi: 10.1128/mcb.00548-06. PubMed PMID: 16894030; PubMed Central PMCID: PMCPmc1636742.

15. Landis GN, Tower J. Superoxide dismutase evolution and life span regulation. Mech Ageing Dev. 2005; 126(3): 365-379. Epub 2005/01/25. doi: 10.1016/j.mad.2004.08.012. PubMed PMID: 15664623.

16. Zelko IN, Mariani TJ, Folz RJ. Superoxide dismutase multigene family: a comparison of the CuZn-SOD (SOD1), Mn-SOD (SOD2), and EC-SOD (SOD3) gene structures, evolution, and expression. Free Radic Biol Med. 2002; 33(3): 337-349. Epub 2002/07/20. PubMed PMID: 12126755.

17. Kwiatowski J, Ayala FJ. Phylogeny of *Drosophila* and related genera: conflict between molecular and anatomical analyses. Molecular phylogenetics and evolution. 1999; 13(2): 319-328. Epub 1999/12/22. doi: 10.1006/mpev.1999.0657. PubMed PMID: 10603260.

18. Bottner CA, Schmidt H, Vogel S, Michele M, Kaufer NF. Multiple genetic and biochemical interactions of Brr2, Prp8, Prp31, Prp1 and Prp4 kinase suggest a function in the control of the activation of spliceosomes in *Schizosaccharomyces pombe*. Curr Genet. 2005; 48(3): 151-161. Epub 2005/09/01. doi: 10.1007/s00294-005-0013-6. PubMed PMID: 16133344.

19. Dolezelova E, Zurovec M, Dolezal T, Simek P, Bryant PJ. The emerging role of adenosine deaminases in insects. Insect Biochem Mol Biol. 2005; 35(5): 381-389. doi: 10.1016/j.ibmb.2004.12.009. PubMed PMID: 15804573.

20. Yoo HS, Seo JH, Yoo JY. CTR9, a component of PAF complex, controls elongation block at the c-Fos locus via signal-dependent regulation of chromatin-bound NELF dissociation. PLoS One. 2013; 8(4): e61055. Epub 2013/04/18. doi: 10.1371/journal.pone.0061055. PubMed PMID: 23593388; PubMed Central PMCID: PMCPmc3623864.

21. Murray AW. Recycling the cell cycle: cyclins revisited. Cell. 2004; 116(2): 221-234. Epub 2004/01/28. PubMed PMID: 14744433.

22. Leclerc V, Tassan JP, O'Farrell PH, Nigg EA, Leopold P. *Drosophila* Cdk8, a kinase partner of cyclin C that interacts with the large subunit of RNA polymerase II. Mol Biol Cell. 1996; 7(4): 505-513. Epub 1996/04/01. PubMed PMID: 8730095; PubMed Central PMCID: PMCPmc275905.

23. Arakane Y, Dixit R, Begum K, Park Y, Specht CA, Merzendorfer H, et al. Analysis of functions of the chitin deacetylase gene family in *Tribolium castaneum*. Insect Biochem Mol Biol. 2009; 39(5-6): 355-365. Epub 2009/03/10. doi: 10.1016/j.ibmb.2009.02.002. PubMed PMID: 19268706.

24. Dixit R, Arakane Y, Specht CA, Richard C, Kramer KJ, Beeman RW, et al. Domain organization and phylogenetic analysis of proteins from the chitin deacetylase gene family of *Tribolium castaneum* and three other species of insects. Insect Biochem Mol Biol. 2008; 38(4): 440-451. Epub 2008/03/18. doi: 10.1016/j.ibmb.2007.12.002. PubMed PMID: 18342249.

25. Zhao Y, Park RD, Muzzarelli RA. Chitin deacetylases: properties and applications. Mar Drugs. 2010; 8(1): 24-46. Epub 2010/02/18. doi: 10.3390/md8010024. PubMed PMID: 20161969; PubMed Central PMCID: PMCPmc2817921.

26. Mukherjee K, Fischer R, Vilcinskas A. Histone acetylation mediates epigenetic regulation of transcriptional reprogramming in insects during metamorphosis, wounding and infection. Front Zool. 2012; 9(1): 25. Epub 2012/10/06. doi: 10.1186/1742-9994-9-25. PubMed PMID: 23035888; PubMed Central PMCID: PMCPmc3538701.

27. Gurevich EV, Gurevich VV. Arrestins: ubiquitous regulators of cellular signaling pathways. Genome Biol. 2006; 7(9): 236.

28. Chang J, Ramasamy S. Molecular-Phylogenetic Characterization of Arrestin-2 From *Maruca vitrata* (Lepidoptera: Crambidae). Annals of the entomological society of America. 2013. doi: http://dx.doi.org/10.1603/AN12136.

29. Pan MH, Du J, Zhang JY, Huang MH, Li T, Cui HJ, et al. Cloning of the flap endonuclease-1 gene in Bombyx mori and identification of an antiapoptotic function. DNA Cell Biol. 2011; 30(10): 763-770. Epub 2011/05/27. doi: 10.1089/dna.2011.1224. PubMed PMID: 21612397.

30. Balakrishnan L, Bambara RA. Flap endonuclease 1. Annu Rev Biochem. 2013; 82: 119-138. Epub 2013/03/05. doi: 10.1146/annurev-biochem-072511-122603. PubMed PMID: 23451868; PubMed Central PMCID: PMCPmc3679248.

31. Kaul G, Pattan G, Rafeequi T. Eukaryotic elongation factor-2 (eEF2): its regulation and peptide chain elongation. Cell Biochem Funct. 2011; 29(3): 227-234. Epub 2011/03/12. doi: 10.1002/cbf.1740. PubMed PMID: 21394738.

32. Danforth BN, Ji S. Elongation factor-1 alpha occurs as two copies in bees: implications for phylogenetic analysis of EF-1 alpha sequences in insects. Molecular biology and evolution. 1998; 15(3): 225-235. Epub 1998/03/21. PubMed PMID: 9501490.

33. Jordal BH. Elongation Factor 1 alpha resolves the monophyly of the haplodiploid ambrosia beetles Xyleborini (Coleoptera: Curculionidae). Insect Mol Biol. 2002; 11(5): 453-465. Epub 2002/09/17. PubMed PMID: 12230544.

34. Regier JC, Shultz JW, Kambic RE. Pancrustacean phylogeny: hexapods are terrestrial crustaceans and maxillopods are not monophyletic. Proceedings Biological sciences / The Royal Society. 2005; 272(1561): 395-401. Epub 2005/03/01. doi: 10.1098/rspb.2004.2917. PubMed PMID: 15734694; PubMed Central PMCID: PMCPmc1634985.

35. Feder ME, Krebs RA. Ecological and evolutionary physiology of heat shock proteins and the stress response in *Drosophila*: complementary insights from genetic engineering and natural variation. Exs. 1997; 83: 155-173. Epub 1997/01/01. PubMed PMID: 9342848.

36. Ohtsubo M, Yoshida T, Seino H, Nishitani H, Clark KL, Sprague GF, Jr., et al. Mutation of the hamster cell cycle gene RCC1 is complemented by the homologous genes of *Drosophila* and *S. cerevisiae*. Embo j. 1991; 10(5): 1265-1273. Epub 1991/05/01. PubMed PMID: 2022190; PubMed Central PMCID: PMCPmc452781.
