# Supplementary material for: Genomic Mining of Phylogenetically Informative Nuclear Markers in Bark and Ambrosia Beetles
Source: PLoS One. 2016 Sep 26;11(9):e0163529. doi: 10.1371/journal.pone.0163529 (PMC5036811; doi:10.1371/journal.pone.0163529)
Supplement: S1 Table — (DOCX) [file pone.0163529.s007.docx]

| **Species code** | **PABP1** | **TPI** | **UBA5** | **IAP2** | **SOD1** | **Prp1** | **ADA2** | **CTR9** | **CCNC** | **Cda4** | **HDAC Rpd3** | **Arr2** | **FEN1** | **EF2** | **Hsp70** | **RCC1** |
| --- | --- | --- | --- | --- | --- | --- | --- | --- | --- | --- | --- | --- | --- | --- | --- | --- |
| BrBre05 | KX160751 | - | KX160704 | - | - | - | - | - | KX160590 | KX160791 | - | - | K160539 | - | KX160690 | - |
| ClLar01 | KX160752 | - | KX160705 | KX160625 | KX160661 | - | - | - | - | - | - | - | - | - | - | - |
| CoPit01 | KX160753 | KX160554 | - | KX160626 | KX160662 | KX160572 | KX160724 | KX160778 | KX160591 |  | KX160738 | KX160641 | K160540 | KX160610 | KX160691 | KX160677 |
| CsMes01 | KX160754 | KX160555 | - | KX160627 | KX160663 | - | - | KX160779 | KX160592 | - | - | - | - | - | - | - |
| CsPse01 | KX160755 | KX160556 | KX160706 | KX160628 | KX160664 | KX160573 | - | - | - | KX160792 | - | KX160642 |  | - | - | KX160678 |
| DiDia03 | KX160756 | - | - | - | - | - | - | - | - | - | KX160739 | KX160643 | - | KX160611 | - | - |
| DiDia04 | KX160757 | KX160557 | KX160707 | KX160629 | - | KX160574 | KX160725 | KX160780 | KX160593 | KX160793 | - | - | K160541 | KX160612 | KX160692 | KX160679 |
| DrDry01 | KX160758 | KX160558 | KX160708 | KX160630 | - | KX160575 | KX160726 | - | KX160594 | - | KX160740 | KX160644 | K160542 | - | - | KX160680 |
| DrOzo02 | KX160759 | KX160559 | - | KX160631 | - | KX160576 | - | - | KX160595 | - | - | KX160645 |  | - | - | KX160681 |
| HlHyl02 | KX160760 | KX160560 | KX160709 | - | KX160665 | KX160577 | - | KX160781 | KX160596 | KX160794 | - | KX160646 | - | - | KX160693 | KX160682 |
| HlKis01 | KX160761 | KX160561 | KX160710 | - | KX160666 | - | KX160727 | KX160782 | KX160597 | KX160795 | KX160741 | - | K160543 | - | KX160694 | - |
| HtHyt06 | KX160762 | KX160562 | KX160711 | - | KX160667 | KX160578 | KX160728 | - | KX160598 | - | KX160742 | KX160647 | K160544 | - | KX160695 | KX160683 |
| IpAca01 | KX160763 | - | KX160712 | KX160632 | - | KX160579 | KX160729 | KX160783 | KX160599 |  | - | KX160648 | K160545 | - | KX160696 | - |
| IpPit03 | KX160764 | KX160563 | KX160713 | KX160633 | KX160668 | KX160580 | KX160730 | - | KX160600 | - | - |  | K160546 | KX160613 | - | KX160684 |
| MoPor01 | KX160765 | - | - | KX160634 | - | - | - | - | KX160601 | - | KX160743 | KX160649 | - | - | KX160697 | - |
| PlPla07 | KX160766 | KX160564 | KX160714 | KX160635 | - | KX160581 | KX160731 | - | KX160602 | KX160796 | KX160744 | KX160650 | - | KX160614 | - | KX160685 |
| PlTri02 | KX160767 | KX160565 | KX160715 | - | KX160669 | KX160582 | KX160732 | - | KX160603 | KX160797 | - | KX160651 | K160547 | KX160615 | - | KX160686 |
| PrPre01 | KX160768 | KX160566 | KX160716 | KX160636 | - | KX160583 | - | KX160784 | - | KX160798 | KX160745 | KX160652 |  | KX160616 | - | KX160687 |
| ScCam02 | KX160769 | - | KX160717 | KX160637 | KX160670 | KX160584 | KX160733 | KX160785 | KX160604 | - | - | KX160653 | K160548 | KX160617 | KX160698 | - |
| ScCne01 | KX160770 | - | KX160718 | - | KX160671 | - | KX160734 | KX160786 | KX160605 | - |  | KX160654 | K160549 | KX160618 | KX160699 | - |
| ScScl02 | KX160771 | - | KX160719 | KX160638 | KX160672 | - | - | - | - | - | - | KX160655 | - | - | - | - |
| ToCha01 | KX160772 | KX160567 | KX160720 | KX160639 | KX160673 | KX160585 | - | KX160787 | KX160606 | KX160799 | KX160746 |  | - | KX160619 | KX160700 | KX160688 |
| ToDen01 | - | - | - | - | - | - | - | - | - | - | - |  | - | KX160620 | KX160701 | - |
| ToDen02 | KX160773 | KX160568 | - | - | - | KX160586 | - | - | KX160607 | - | - | KX160656 | K160550 | - | - | KX160689 |
| ToTom01 | KX160774 | KX160569 | KX160721 | - | - | KX160587 | - | KX160788 | - | KX160800 | KX160747 | KX160657 | K160551 | KX160621 | - | - |
| TsCha02 | KX160775 | - | - | - | KX160674 | KX160588 | KX160735 | - | - | KX160801 | KX160748 | KX160658 | - | KX160622 | KX160702 | - |
| XyXyl00 | KX160776 | KX160570 | KX160722 | KX160640 | KX160675 | KX160589 | KX160736 | KX160789 | KX160608 | KX160802 | KX160749 | KX160659 | K160552 | KX160623 | KX160703 | - |
| XyXyl03 | KX160777 | KX160571 | KX160723 | - | KX160676 | - | KX160737 | KX160790 | KX160609 | KX160803 | KX160750 | KX160660 | K160553 | KX160624 | - | - |
