# Supplementary material for: Genomic Mining of Phylogenetically Informative Nuclear Markers in Bark and Ambrosia Beetles
Source: PLoS One. 2016 Sep 26;11(9):e0163529. doi: 10.1371/journal.pone.0163529 (PMC5036811; doi:10.1371/journal.pone.0163529)
Supplement: S2 Table — For each of the 16 genes, the proportion of different nucleotide sites between sequences was calculated. The most frequently PCR amplified species (Xyleborus affinis) was compared with members of the other tribes and subfamilies and the lower value was reported. PIC = Parsimony informative characters, HI = Homoplasy index and RI = Retention index. (DOCX) [file pone.0163529.s008.docx]

| **Gene** | **Xyleborini + Dryocoetini** | | | | **Ipini + Premnobini** | | | | **Hylurgini** | | |  | **Scolytini** | | | | **Other Curculionidae** | | | | **Platipodinae** | | | | **PIC** | **HI** | **RI** |
| --- | --- | --- | --- | --- | --- | --- | --- | --- | --- | --- | --- | --- | --- | --- | --- | --- | --- | --- | --- | --- | --- | --- | --- | --- | --- | --- | --- |
|  | **1st** | **2nd** | **3rd** | **Total** | **1st** | **2nd** | **3rd** | **Total** | **1st** | **2nd** | **3rd** | **Total** | **1st** | **2nd** | **3rd** | **Total** | **1st** | **2nd** | **3rd** | **Total** | **1st** | **2nd** | **3rd** | **Total** |  |  |  |
| ***PABP1*** | 0.15 | 0 | 0.076 | 0.029 | 0.023 | 0 | 0.42 | 0.14 | 0.031 | 0.016 | 0.429 | 0.151 | 0.023 | 0.016 | 0.521 | 0.177 | 0.015 | 0.008 | 0.42 | 0.146 | 0.023 | 0.008 | 0.471 | 0.159 | 162 | 0.7404 | 0.3857 |
| ***TPI*** | 0.032 | 0.016 | 0.13 | 0.061 | 0.129 | 0.016 | 0.574 | 0.235 | 0.081 | 0 | 0.463 | 0.173 | - | - | - | - | 0.081 | 0.016 | 0.574 | 0.218 | 0.113 | 0.048 | 0.5 | 0.207 | 255 | 0.5904 | 0.4137 |
| ***UBA5*** | 0.009 | 0.009 | 0.121 | 0.046 | 0.138 | 0.036 | 0.505 | 0.223 | 0.101 | 0.036 | 0.533 | 0.211 | 0.194 | 0.054 | 0.598 | 0.248 | 0.119 | 0.063 | 0.551 | 0.26 | 0.156 | 0.045 | 0.542 | 0.275 | 168 | 0.6353 | 0.4054 |
| ***Iap2*** | 0.041 | 0.013 | 0.19 | 0.073 | 0.123 | 0.067 | 0.379 | 0.175 | 0.205 | 0.067 | 0.5 | 0.277 | 0.151 | 0.04 | 0.552 | 0.238 | 0.137 | 0.053 | 0.483 | 0.204 | 0.288 | 0.213 | 0.5 | 0.32 | 379 | 0.5711 | 0.3352 |
| ***SOD1*** | 0.014 | 0.056 | 0.048 | 0.039 | 0.071 | 0.099 | 0.54 | 0.23 | 0.086 | 0.07 | 0.476 | 0.196 | 0.114 | 0.07 | 0.603 | 0.25 | 0.1 | 0.099 | 0.556 | 0.235 | 0.143 | 0.127 | 0.556 | 0.279 | 133 | 0.609 | 0.4014 |
| ***Prp1*** | 0.045 | 0.014 | 0.18 | 0.069 | 0.06 | 0.014 | 0.5 | 0.159 | 0.06 | 0.014 | 0.52 | 0.164 | 0.06 | 0.014 | 0.68 | 0.206 | 0.045 | 0.014 | 0.5 | 0.153 | 0.045 | 0.014 | 0.52 | 0.159 | 218 | 0.615 | 0.4099 |
| ***ADA2*** | 0.017 | 0.048 | 0.115 | 0.052 | 0.051 | 0.048 | 0.538 | 0.179 | 0.068 | 0.065 | 0.577 | 0.208 | 0.085 | 0.032 | 0.577 | 0.22 | - | - | - | - | 0.068 | 0.048 | 0.5 | 0.191 | 239 | 0.5025 | 0.4369 |
| ***Ctr9*** | 0 | 0 | 0.065 | 0.021 | 0.098 | 0.017 | 0.491 | 0.199 | 0.027 | 0.017 | 0.426 | 0.163 | 0.08 | 0.043 | 0.444 | 0.184 | 0.045 | 0.009 | 0.5 | 0.178 | - | - | - | - | 222 | 0.5138 | 0.4124 |
| ***CC-*** | 0 | 0 | 0.154 | 0.047 | 0.018 | 0 | 0.596 | 0.193 | 0.053 | 0 | 0.577 | 0.199 | 0.053 | 0.016 | 0.596 | 0.205 | 0.088 | - | 0.558 | 0.199 | 0.123 | 0.016 | 0.654 | 0.251 | 168 | 0.6596 | 0.4256 |
| ***Cda4*** | 0 | 0 | 0.05 | 0.015 | 0 | 0.077 | 0.7 | 0.235 | 0 | 0 | 0.35 | 0.132 | - | - | - | - | 0 | 0 | 0.55 | 0.162 | 0.045 | 0.038 | 0.35 | 0.191 | 156 | 0.5449 | 0.4587 |
| ***HDAC Rpd3*** | 0.009 | 0.014 | 0.149 | 0.056 | 0.052 | 0 | 0.522 | 0.185 | 0.057 | 0 | 0.532 | 0.192 | - | - | - | - | 0.047 | 0 | 0.522 | 0.184 | 0.08 | 0.009 | 0.498 | 0.217 | 282 | 0.5803 | 0.39 |
| ***Arr2*** | 0.035 | 0 | 0.154 | 0.085 | 0.036 | 0.071 | 0.423 | 0.171 | 0 | 0.036 | 0.577 | 0.207 | 0 | 0.036 | 0.5 | 0.232 | 0 | 0.036 | 0.538 | 0.207 | 0.214 | 0.036 | 0.654 | 0.305 | 239 | 0.605 | 0.4318 |
| ***FEN1*** | 0.035 | 0.009 | 0.18 | 0.074 | 0.159 | 0.069 | 0.505 | 0.247 | 0.159 | 0.078 | 0.505 | 0.262 | 0.212 | 0.103 | 0.559 | 0.291 | 0.248 | 0.069 | 0.613 | 0.306 | 0.265 | 0.121 | 0.631 | 0.335 | 197 | 0.5713 | 0.4267 |
| ***EF2*** | 0.019 | 0 | 0.212 | 0.075 | 0.019 | 0 | 0.385 | 0.131 | 0.037 | 0 | 0.269 | 0.1 | 0.037 | 0 | 0.404 | 0.144 | - | - | - | - | 0.056 | 0 | 0.404 | 0.15 | 192 | 0.536 | 0.5189 |
| ***Hsp70*** | - | - | - | - | 0.048 | 0 | 0.496 | 0.178 | 0.048 | 0 | 0.454 | 0.171 | 0.048 | 0.007 | 0.433 | 0.159 | 0.048 | 0.007 | 0.426 | 0.155 | 0.102 | 0.007 | 0.539 | 0.212 | 185 | 0.5215 | 0.4236 |
| ***RCC1*** | - | - | - | - | 0.041 | 0.042 | 0.532 | 0.194 | 0.061 | 0.021 | 0.489 | 0.194 | - | - | - | - | 0.082 | 0.042 | 0.574 | 0.222 | 0.082 | 0.063 | 0.553 | 0.229 | 127 | 0.5031 | 0.4302 |
