# Supplementary material for: Genomic Mining of Phylogenetically Informative Nuclear Markers in Bark and Ambrosia Beetles
Source: PLoS One. 2016 Sep 26;11(9):e0163529. doi: 10.1371/journal.pone.0163529 (PMC5036811; doi:10.1371/journal.pone.0163529)
Supplement: S3 Table — The main problems for further development are reported, together with data on fragment length, and number and length of introns for 18 of these markers. The same information could not be derived for markers with low number of sequences. (DOCX) [file pone.0163529.s009.docx]

| **Gene** | **N sequences, PCR and sequencing details** | **Lenght of the fragment and intron number** |
| --- | --- | --- |
| *actin beta* | 18 – Low quality sequences - paralogs? | 573bp - No intron |
| *chromosome X open reading frame 56* | 14 - Unspecific PCR amplification - Indels - paralogs? | 408bp - 3 introns - 1 Indel (HtHyt06) |
| *eukaryotic release factor 1* | 15 - Short exon fragments | 282bp - 1 intron |
| *splicing factor U2F* | 13 - No PCR amplification for Platypodinae | 390bp - 2 introns absent in Scolytini |
| *methylmalonate semialdeyde dehydroenase* | 13 - PCR amplification for different groups - Short fragment | 300bp - 1 intron, present in all the species |
| *muscular protein 20* | 12 - Short exon fragments | 280bp - 1 intron, present in all the species |
| *elongation initiation factor 5C* | 13 - Short exon fragment | <200bp |
| *phosphatidylinositol 4-kinase type 2-alpha* | 10 - No PCR amplification in Scolytini tribe and Platypodinae | 603bp - 3 introns |
| *Gelsolin* | 10 – Short exon fragment | <250bp |
| *C-1-tetrahydrofolate synthase* | 10 - High PCR amplification success for Hylurgini | 480bp - 1 intron |
| *Alpha-spectrin* | 9 - No PCR amplification in Scolytini, Ipini and Hylurgini | 339bp - 3 introns |
| *alanyl-tRNAsynthetase* (*AATS*) | 9 - General low PCR amplification | 492bp - 3 introns |
| *heat shock protein 90* | 7 - No PCR amplification in non-Scolytinae beetles | 1212bp - No intron - Indels |
| *dihydrolipoamide dehydrogenase E3* | 7 - Low PCR amplification in non-Scolytinae beetles | 300bp - 2 introns |
| *mannose-1-phosphate guanyltransferase* α (*Mpgt*) | 6 - High PCR amplification only in Hylurgini | 597bp - No introns - Indels |
| *Na+/K+ ATPase alpha subunit* | 5 - PCR amplification in Dryocoetini and Ipini | 636bp -1 intron |
| *F-box only protein 11* | 5 - General low PCR amplification | 465bp - 2 short intron |
| *uracil-DNA degrading factor* | 5 - High PCR amplification for Hylurgini, unspecific amplification | 417bp - 1 intron - indels |
| *6-phosphogluconate dehydrogenase* | 4 - Unspecific amplification (fungi, bacteria, nematodes) |  |
| *glicoside hydrolase family 31* | 11 - Multiple copies - unalignable regions |  |
| *odorant-binding protein* | 8 - Multiple copies - unalignable regions |  |
| *GTP binding protein* | 3 |  |
| *cathepsinL* | 1 |  |
| *troponin C* | 2 |  |
| *glucose-6-phosphate isomerase* (*PGI*) | 8 - Unspecific PCR amplification from bacteria |  |
| *Acetyl coenzima A synthetase* | 1 |  |
| *Uridine cytidine kinase* | 1 |  |
| *Prophenoloxidase* (*PPO*) | 2 - Ambiguous BLAST characterization |  |
| *pre-mRNA-processing factor 6* (*prp6*) | 1 |  |
| *Maxillopedia* (*mxp*) | 1 |  |
| *nuclear protein localization protein 4* | 3 |  |
| *calmodulin1* | 1 |  |
| *syntaxin-1A* (*STX1A*) | 4 - Short fragment <300bp |  |
| *transmembrane protein 120B* | 2 |  |
| *chitin synthase* | 2 - Ambiguous BLAST characterization (CHS1/CHS2) - 1300 bp |  |
| *ATP-dependent RNA helicase DDX49* | 3 |  |
| *general transcription factor IIH subunit 3* | 2 |  |
| *translation initiation factor 3 subunit L (IF3)* | 2 |  |
| *eukaryotic translation initiation factor 6* | 1 |  |
| *isocitrate dehydrogenase* (*IDH*) | 3 |  |
| *ecdysone receptor* (*Ecr*) | 1 |  |
